# Efficient privacy-preserving variable-length substring match for genome sequence

**DOI:** 10.1186/s13015-022-00211-1

**Published:** 2022-04-26

**Authors:** Yoshiki Nakagawa, Satsuya Ohata, Kana Shimizu

**Affiliations:** 1 Department of Computer Science and Engineering, Waseda University, Tokyo, Japan; 2 Self-employment, Tokyo, Japan; 3 National Institute of Advanced Industrial Science and Technology, Tokyo, Japan

**Keywords:** Private genome sequence search, Secure multiparty computation, Secret sharing, FM-index, Suffix array, LCP array, Maximal exact match

## Abstract

The development of a privacy-preserving technology is important for accelerating genome data sharing. This study proposes an algorithm that securely searches a variable-length substring match between a query and a database sequence. Our concept hinges on a technique that efficiently applies FM-index for a secret-sharing scheme. More precisely, we developed an algorithm that can achieve a secure table lookup in such a way that $V[V[\ldots V[p_0] \ldots ]]$ is computed for a given depth of recursion where $p_0$ is an initial position, and *V* is a vector. We used the secure table lookup for vectors created based on FM-index. The notable feature of the secure table lookup is that time, communication, and round complexities are not dependent on the table length *N*, after the query input. Therefore, a substring match by reference to the FM-index-based table can also be conducted independently against the database length, and the entire search time is dramatically improved compared to previous approaches. We conducted an experiment using a human genome sequence with the length of 10 million as the database and a query with the length of 100 and found that the query response time of our protocol was at least three orders of magnitude faster than a non-indexed database search protocol under the realistic computation/network environment.

## Introduction

The dramatic reduction in the cost of genome sequencing has prompted increased interest in personal genome sequencing over the last 15 years. Extensive collections of personal genome sequences have been accumulated both in academic and industrial organizations, and there is now a global demand for sharing the data to accelerate scientific research [1, 2]. As discussed in previous studies, disclosing personal genome information has a high privacy risk [3], so it is crucial to ensure that individuals’ privacy is protected upon data sharing. At present, the most popular approach for this is to formulate and enforce a privacy policy, but it is a time-consuming process to reach an agreement, especially among stakeholders with different legal backgrounds, which slows down the pace of research. Therefore, there is a strong demand for privacy-preserving technologies that can potentially compensate for or even replace the traditional policy-based approach [4, 5]. One important application that needs a privacy-preserving technology is private genome sequence search, where different stakeholders respectively hold a query sequence and a database sequence and the goal is to let the query holder know the result while simultaneously keeping the query and the database private. Many studies have addressed the problem of how to compute exact or approximate edit distance or the longest common substring (LCS) through techniques based on homomorphic encryption [6–8] and secure multi-party computation (MPC) [9–15], or how to compute sequence similarity based on private set intersection [16]. While these studies can evaluate global sequence similarity for two sequences of similar length, other studies address the problem of finding a substring between a query and a long genome sequence or a set of long genome sequences, with the aim of evaluating local sequence similarity [17–23]. Shimizu et al. proposed an approach to combine an additive homomorphic encryption and index structures such as FM-index [24] and the positional Burrows-Wheeler transform [25] to find the longest prefix of a query that matches a database (LPM) and a set-maximal match for a collection of haplotypes [17]. Sudo et al. used a similar approach and improved the time and communication complexities for LPM on a protein sequence by using a wavelet matrix [19]. Ishimaki et al. improved the round complexity of a set-maximal match, though the search time was more than one order of magnitude slower than [17] due to the heavy computational cost caused by the fully homomorphic encryption [18]. Sotiraki et al. used the Goldreich-Micali-Wigderson protocol to build a suffix tree for a set-maximal match [20]. According to experiments by [21], the search time of [20] is one order of magnitude slower than [17, 21]. Mahdi et al. [21] used a garbled circuit to build a suffix tree for substring match and a set-maximal match under a different security assumption such that the tree-traversal pattern is leaked to the cloud server. Chen et al. [22] and Popic et al. [23] found fixed-length substring matches using a one-way hash function or homomorphic encryption on a public cloud under a security assumption such that the database is a public sequence and a query is leaked to a private cloud server.

In this study, we aim to improve privacy-preserving substring match under the security assumption such that both the query and the database sequence are strictly protected. We first propose a more efficient method for finding LPM, and then extend it to find the longest maximal exact match (LMEM), which is more practically important in bioinformatics. We designed the protocol for LMEM for ease of explanation, and the protocol can be applied to similar problems such as finding all maximal exact matches (MEMs) with a small modification. To our knowledge, this is the first study to address the problem of securely finding MEMs.

### Our contribution

The time complexity of the previous studies [17, 19] include the factor of $N$, and thus they do not scale well to a large database. For a similar reason, using secure matching protocols (e.g., [26]) for the shares (or tags in searchable encryption) of all substrings in a query and database is even worse in terms of time complexity. To achieve a real-time search on an actual genome database, we propose novel secret-sharing-based protocols that do not include the factor of $N$ in the time, communication, and round complexities for the search time (i.e., the time after the input of a query until the end of the search).

The basic idea of the protocols is to represent the database string by a compressed index [24, 27] and store the index as a lookup table. LPM and MEMs are found by at most $\ell$ and $2\ell$ table lookups respectively, where $\ell$ is the length of the query. More specifically, the table $V$ is referenced in a recursive manner; i.e., one needs to obtain $V[j]$, where $j=V[i]$, given *i*. To ensure security, we need to compute $V[j]$ without seeing any element of $V$. The key technical contribution of this study is an efficient protocol that achieves this type of recursive reference. We named the protocol secret-shared recursive oblivious transfer (ss-ROT). While the previous studies require $O(N)$ time complexity to ensure security, the time, communication, and round complexities of ss-ROT are all $O(\ell )$ for $\ell$ recursive table lookups, except for the preparation of the table and generation of shares before the query input. Since the entire protocols mainly consist of $\ell$ table lookups for LPM, and $2\ell$ table lookups and $2\ell$ inner product computations for LMEM, the search times for LPM and LMEM do not depend on the database size. In addition to the protocols based on ss-ROT, we developed a protocol to reduce data transfer size in the initial step by using a similar approach taken in ss-ROT. The protocol offers a reasonable trade-off between the amount of reduction in data transfer in the initial step and the increase in computational cost in the later step.

We implemented the proposed protocol and tested it on substrings of a human genome sequence $10^3$ to $10^7$ in length and confirmed that the actual CPU time and data transfer overhead were in good agreement with the theoretical complexities. We also found that the search time of our protocol was three orders of magnitude faster than that of the previous method [17, 19]. For conducting further performance analysis, we designed and implemented baseline protocols using major techniques of secret-sharing-based protocols. The results showed that the search times of our protocols were at least two orders of magnitude faster than those of the baseline protocols.

## Preliminaries

### Secure computation based on secret sharing

**Fig. 1:**
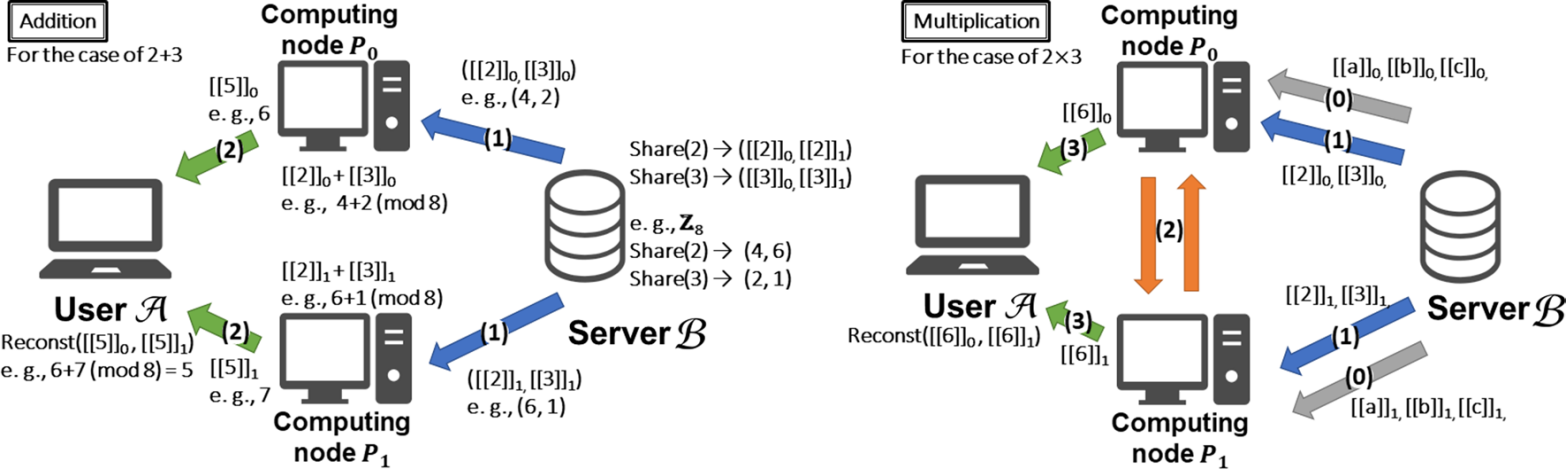
Arithmetic addition and multiplication over secret sharing

Here, we explain the 2-out-of-2 additive secret sharing ((2, 2)-SS) scheme and how to securely compute arithmetic/Boolean gates (Fig. 1).

*Secret sharing and secure computation* In *t*-out-of-*n* secret sharing (e.g., [28]), we split the secret value *x* into *n* pieces, and can reconstruct *x* by combining more or an equal number of *t* pieces. We call the split pieces “share”. The basic security notion for secret sharing is that we cannot obtain any information about *x* even if we gather less than or equal to $(t-1)$ shares. In this paper, we consider a case with $(t,n) = (2,2)$. A 2-out-of-2 secret sharing ((2, 2)-SS) scheme over $\mathbb {Z}_{2^n}$ consists of two algorithms: $\mathsf {Share}$ and $\mathsf {Reconst}$. $\mathsf {Share}$ takes as input $x \in \mathbb {Z}_{2^n}$ and outputs $([\![x]\!]_0, [\![x]\!]_1) \in \mathbb {Z}_{2^n}^2$, where the bracket notation $[\![x]\!]_i$ denotes the arithmetic share of the *i*-th party (for $i \in \{0,1\}$). We denote $[\![x]\!] = ([\![x]\!]_0,[\![x]\!]_1)$ as their shorthand. $\mathsf {Reconst}$ takes as inputs $[\![x]\!]_0$ and $[\![x]\!]_1$ and outputs *x*. For arithmetic sharing $[\![x]\!]_i$ and Boolean sharing $[\![x]\!]^B_i$, we consider power-of-two integers *n* (e.g., $n=16$) and $n=1$, respectively.

Depending on the secret sharing scheme, we can compute arithmetic/Boolean gates over shares; that is, we can execute some kind of processing related to *x* without *x*. This means it is possible to perform some computation without violating the privacy of the secret data, and is called secure (multi-party) computation. It is known that we can execute arbitrary computation by combining basic arithmetic/Boolean gates. In the following paragraphs, we show how to concretely compute these gates over shares.

**Table 1:** Secure subprotocols used in this paper

	Input	Output
$\mathsf {Equality}$	$[\![x]\!]$, $[\![y]\!]$	$[\![z]\!]^B$ s.t. $z = 1$ if $x = y$ otherwise $z = 0$
$\mathsf {Comp}$	$[\![x]\!]$, $[\![y]\!]$	$[\![z]\!]^B$ s.t. $z = 1$ if $x < y$ otherwise $z = 0$
$\mathsf {CastUp}$	$[\![x]\!] \in \mathbb {Z}_{2^n}$, $n'$	$[\![x]\!] \in \mathbb {Z}_{2^{n'}}$ ($n < n'$)
$\mathsf {B2A}$	$[\![x]\!]^B$	$[\![x]\!]$
$\mathsf {Choose}$	$[\![x]\!]$, $[\![y]\!]$, $[\![e \in \{0,1\}]\!]$	$[\![z]\!]$ s.t. $z = x$ if $e = 1$, otherwise ($e = 0$) $z = y$

*Semi-honest secure two-party computation based on* (2, 2)-*Additive SS* We use a standard (2, 2)-additive SS scheme, defined by

- $\mathsf {Share}(x):$ randomly choose $r \in \mathbb {Z}_{2^n}$ and let $[\![x]\!]_0 = r$ and $[\![x]\!]_1 = x - r$.
- $$\mathsf {Reconst}([\![x]\!]_0, [\![x]\!]_1):$$ output $$[\![x]\!]_0 + [\![x]\!]_1$$.

Note that one of the shares of *x* ($[\![x]\!]_0$ or $[\![x]\!]_1$) does not reveal any information about *x*. In Fig. 1, the secret value $x = 2$ is split into $[\![x]\!]_0 = 4$ and $[\![x]\!]_1 = 6$. These are valid (2, 2)-additive shares because $4 + 6 \equiv 2 \pmod 8$ holds. Even if we can see $[\![x]\!]_0 = 4$, we cannot decide the value of *x* since we execute a split of *x* uniformly at random. This means, in Fig. 1, computing nodes $P_0$ and $P_1$ cannot obtain any information about *x* as long as these two nodes do not collude. On the other hand, we can compute arithmetic $\textsf {ADD}/\textsf {MULT}$ gates over shares as follows:

- $[\![z]\!] \leftarrow \textsf {ADD}([\![x]\!], [\![y]\!])$ can be done locally by just adding each party’s share on *x* and on *y*. In Fig. 1 (left), we show an example of secure addition. $P_0/P_1$ obtain shares 6/7 by adding their two shares. In this process, $P_0/P_1$ cannot find they are computing $2+3$.
- Multiplication is more complex than addition. There are various methods for multiplication over shares, most of which require communication between computing nodes. In this paper, we use the standard method for $[\![w]\!] \leftarrow \textsf {MULT}([\![x]\!], [\![y]\!])$ based on Beaver triples (BT) [29]. Such a triple consists of $\mathsf {bt}_0 = (a_0, b_0, c_0)$ and $\mathsf {bt}_1 = (a_1, b_1, c_1)$ such that $(a_0 + a_1)(b_0 + b_1) = (c_0 + c_1)$. Hereafter, *a*, *b*, and *c* denote $a_0 + a_1$, $b_0 + b_1$, and $c_0 + c_1$, respectively. We use these BTs as auxiliary inputs for computing $\textsf {MULT}$. Note that we can compute them in advance (or in offline phase) since they are independent of inputs $[\![x]\!]$ and $[\![y]\!]$. We adopt a trusted initializer setting (e.g., [30, 31]); that is, BTs are generated by the party other than two computing nodes and then distributed. In the online phase of $\textsf {MULT}$, each *i*-th party $P_i$ ($i \in \{0,1\}$) can compute the multiplication share $[\![z]\!] = [\![xy]\!]$ as follows:

1. $P_i$ first computes $([\![x]\!]_i - a_i)$ and $([\![y]\!]_i - b_i)$, and sends them to $P_{1-i}$.
2. $$P_i$$ reconstructs $$x'= x - a$$ and $$y' = y - b$$.
3. $P_0$ computes $[\![z]\!]_0 = x'y' + x'b_0 + y'a_0 + c_0$, and $P_1$ computes $[\![z]\!]_1 =x'b_1 + y'a_1 + c_1$.
  Here, $[\![z]\!]_0$ and $[\![z]\!]_1$ calculated with the above procedures are valid shares of *xy*; that is, $\mathsf {Reconst}([\![z]\!]_0, [\![z]\!]_1) = xy$. We shorten the notations and write the $\textsf {ADD}$ and $\textsf {MULT}$ protocols simply as $[\![x]\!] + [\![y]\!]$ and $[\![x]\!] \cdot [\![y]\!]$, respectively.

We also write $\textsf {ADD}(\textsf {ADD}([\![x_\mathrm{A}]\!], [\![x_\mathrm{B}]\!]), [\![x_\mathrm{C}]\!])$ as $\Sigma _{c=\{ \mathrm{A}, \mathrm{B}, \mathrm{C} \}} [\![x_c]\!]$. Note that, similarly to the $\textsf {ADD}$ protocol, we can also locally compute multiplication by constant *c*, denoted by $c \cdot [\![x]\!]$. We can easily extend the above protocols to Boolean gates. By converting $+$ and − into $\oplus$ in the arithmetic $\textsf {ADD}$ and $\textsf {MULT}$ protocols, we can obtain the $\textsf {XOR}$ and $\textsf {AND}$ protocols, respectively. We can construct $\textsf {NOT}$ and $\textsf {OR}$ protocols from the properties of these gates. When we compute $\textsf {NOT}([\![x]\!]^B_0, [\![x]\!]^B_1)$, $P_0$ and $P_1$ output $\lnot [\![x]\!]^B_0$ and $[\![x]\!]^B_1$, respectively. When we compute $\textsf {OR}([\![x]\!]^B, [\![y]\!]^B)$, we compute $\lnot \textsf {AND}(\lnot [\![x]\!]^B, \lnot [\![y]\!]^B)$. We shorten the notations and write $\textsf {XOR}$, $\textsf {AND}$, $\textsf {NOT}$, and $\textsf {OR}$ simply as $[\![x]\!] \oplus [\![y]\!]$, $[\![x]\!] \wedge [\![y]\!]$, $\lnot [\![x]\!]$, and $[\![x]\!] \vee [\![y]\!]$, respectively. By combining the above gates, we can securely compute higher-level protocols. The functionality of the secure subprotocols [15] used in this paper are shown in Table 1. Due to space limits, we omit the details of their construction. Note that we can compute $\mathsf {Choose}$ by $[\![z]\!] = [\![y]\!] + [\![e]\!] \cdot ([\![x]\!] - [\![y]\!])$. In this paper, we consider the standard simulation-based security notion in the presence of semi-honest adversaries (for 2PC), as in [32]. We show the definition in Appendix 2. Roughly speaking, this security notion guarantees the privacy of the secret under the condition that computing nodes do not deviate from the protocol; that is, although computing nodes are allowed to execute arbitrary attacks in their local, they do not (maliciously) manipulate transmission data to other parties. The building blocks we adopt in this paper satisfy this security notion. Moreover, as described in [32], the composition theorem for the semi-honest model holds; that is, any protocol is privately computed as long as its subroutines are privately computed.

### Index structure for string search

*Notation and definition* $\Sigma$ denotes a set of ordered symbols. A string consists of symbols in $\Sigma$. We denote a lexicographical order of two strings *S* and $S'$ by $S \le S'$ (i.e., A < C < G < T and AAA < AAC). We denote the *i*-th letter of a string *S* by *S*[*i*] and a substring starting from the *i*-th letter to the *j*-th letter by *S*[*i*, *j*]. The index starts with 0. The length of *S* is denoted by |*S*|. A reverse string of *S* (i.e., $S[|S|-1],\ldots ,S[0]$) is denoted by $\hat{S}$. We consider a direction from the *i*-th position to the *j*-th position as rightward if $i < j$ and leftward otherwise.

Given a query $w$ and a database *S*, we define the longest prefix that matches a database string (LPM) by $\max _{(0, j)}\{ j | w[0,\ldots ,j] = S[k,\ldots ,l] \}$, where $0 \le j < \ell$ and $0 \le k \le l< N$, and the longest maximal exact match (LMEM) by $\max _{(i, j)}\{ j-i | w[i,\ldots ,j] = S[k,\ldots ,l] \}$, where $0 \le i \le j < \ell$ and $0 \le k \le l< N$.

*FM-Index and related data structures* FM-Index [24] and related data structures [27] are widely used for genome sequence search. Given a query string $w$ of length $\ell$ and a database string *S* of length *N*, [24] enables LPM to be found in $O(\ell )$ time regardless of *N*, and it also enables LMEM to be found in $O(\ell )$ if auxiliary data structures are used [27]. Given all the suffixes of a string *S*: $S[0,\ldots ,|S|-1]$, $S[1,\ldots ,|S|-1], \ldots , S[|S|-1]$, a suffix array is an array of positions $(p_0, \ldots , p_{|S|-1})$ such that $S[p_0,\ldots ,|S|-1] \le S[p_1,\ldots ,|S|-1] \le S[p_2,\ldots , |S|-1], \ldots , \le S[p_{|S|-1},\ldots , |S|-1]$. We denote the suffix array of *S* by *SA* and denote its *i*-th element by *SA*[*i*]. A Burrows-Wheeler transform (BWT) is a permutation of the sequence *S* such that its *i*-th letter becomes $S[SA[i] - 1]$. We denote a BWT of *S* by *L* and denote its *i*-th letter by *L*[*i*]. Let us define a rank of *S* for a letter $c\in \Sigma$ at position *t* by $\mathsf {Rank}_{c}(t,S) = |\{ j | S[j]=c, 0\le j < t \} |$ and a count of occurrences of letters that are lexicographically smaller than *c* in *S* by $\mathsf {CF}_{c}(S) = \sum _{r < c} \mathsf {Rank}_{r}(|S|,S)$, and the operation $\mathsf {LF}_{c}(i, S) = \mathsf {CF}_{c}(L) + \mathsf {Rank}_{c}(i, L)$. The match between $w$ and *S* is reported as a form of left-closed and right-open interval on *SA*, and the lower and upper bounds of the interval are respectively computed by $\mathsf {LF}$. Given a letter *c* and an interval [*f*, *g*) that corresponds to suffixes that share the prefix *x* (i.e., [*f*, *g*) reports the locations of the substring *x* in *S*), we can find a new interval that corresponds to all suffixes that share the prefix *cx* (i.e., locations of the substring *cx*) by

1 $$\begin{aligned}{}[f', g') = [\mathsf {LF}_c(f, S), \mathsf {LF}_c(g, S) ). \end{aligned}$$

The leftward extension of the match is called a backward search, which is the main functionality of FM-Index. By starting the search with the initial interval [0, *N*) and conducting the backward searches for $w[\ell -1], w[\ell -2], \ldots$, the longest suffix match is detected when $f=g$. $\mathsf {Rank}$ and $\mathsf {CF}$ are precomputed and stored in an efficient from that can be searched in constant time. Therefore, the longest suffix match can be computed in $O(\ell )$ time. LPM is found if the search is conducted on $\hat{S}$ and match is extended by $w[0], w[1], \ldots , w[\ell -1]$.

Searching LMEM by repeating LPM for $$w[0, \ldots , \ell -1], w[1,\ldots , \ell -1], w[2,\ldots , \ell -1], \ldots , w[\ell -1]$$ takes $$O(\ell ^2)$$ time. We can improve it to $$O(\ell )$$ time by using the longest common prefix (LCP) array and related data structures [27]. The LCP array, denoted by $$\mathsf {LCP}$$, is an array that stores the length of the longest prefix of $$S[\mathsf {SA}[i-1] , |S|-1]$$ and $$S[\mathsf {SA}[i] , |S|-1]$$ in $$\mathsf {LCP}[i]$$ for $$0 < i \le N$$. The lcp-interval [*i*, *j*) of lcp-value *d* is an interval such that it satisfies $$\mathsf {LCP}[i]<d$$, $$\mathsf {LCP}[j]<d$$, $$\mathsf {LCP}[k] > d$$ for all $$k\in \{i+1,\ldots , j-1\}$$, and $$\mathsf {LCP}[k]=d$$ for at least one $$k\in \{i+1,\ldots , j-1\}$$, and is denoted by $$d-[i,j)$$. $$d-[i,j)$$ corresponds to all the suffixes that share the prefix $$S[SA[i],\ldots , SA[i]+d-1]$$. The parent interval of $$d-[i,j)$$ is the lcp-interval $$h-[m, n)$$ such that $$h<d$$ and $$0 \le m \le i< j \le n <N$$, and there is no other lcp-interval $$t-[r, s)$$ such that $$h<t<d$$ and $$0 \le m \le r \le i< j \le s \le n <N$$. The parent of the lcp-interval [*f*, *g*) can be found by

2 $$\begin{aligned}{}[f', g')= {\left\{ \begin{array}{ll} [\mathsf {PSV}[f_i], \mathsf {NSV}[f_i]) &{} \mathsf {LCP}[g_i] \le \mathsf {LCP}[f_i] \\ {[}\mathsf {PSV}[g_i], \mathsf {NSV}[g_i]) &{} (otherwise), \end{array}\right. } \end{aligned}$$

where $\mathsf {PSV}[i] = \max \{ j | 0 \le j< i \wedge \mathsf {LCP}[j] < \mathsf {LCP}[i] \}$ and $\mathsf {NSV}[i] = \min \{ j | i \le j< N \wedge \mathsf {LCP}[j] < \mathsf {LCP}[i] \}$. By finding a parent interval using $\mathsf {PSV}$ and $\mathsf {NSV}$ whenever it fails to extend the match, we can avoid useless backward searches, and thus LMEM is found at most $2\ell$ backward searches. $\mathsf {LCP}$, $\mathsf {PSV}$ and $\mathsf {NSV}$ are precomputed and stored in an efficient form that can be searched in constant time, so we can find LMEM in $O(\ell )$ time. See section 5.2 of [27] for more details of the data structures. Examples of the search by FM-Index, $\mathsf {LCP}$, $\mathsf {PSV}$, and $\mathsf {NSV}$ are provided in Appendix 1.

**Fig. 2:**
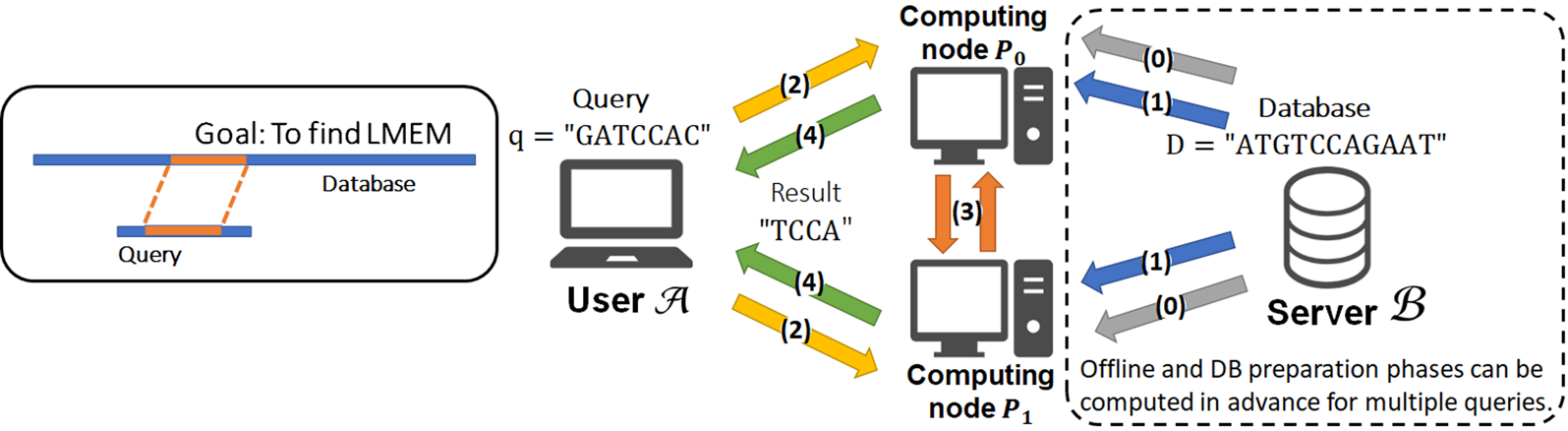
Schematic view of our goal and model. (0) Server (DB holder) distributes Beaver triples. (A reliable third party can serve as the trusted initializer instead.) (1) Server distributes shares of the database. (2) User (query holder) distributes shares of the query. (3) The computing nodes jointly calculate shares of the result. (4) The results are sent to User. The offline phase is (0), DB preparation phase is (1), and Search phase consists of (2)–(4)

**Table 2:** Summary of complexities for our protocols and related protocols

	Btime	Bsize	Dtime	Dsize	Stime	Comm.	Round
ss-ROT (proposed)	0	0	$\ell N$	$\ell N$	$\ell$	$\ell$	$\ell$
Secure LPM (proposed)	$\ell$	$\ell$	$\ell N$	$\ell N$	$\ell$	$\ell$	$\ell$
[17, 19] (LPM by AHE)	−	−	−	−	$\ell N$	$\ell \sqrt{N}$	$\ell$
Baseline LPM	$\ell ^2 N$	$\ell ^2 N$	*N*	*N*	$\ell ^2 N$	$\ell ^2 N$	$\log \ell +\log N$
Secure LMEM (proposed)	$\ell ^2$	$\ell ^2$	$\ell N$	$\ell N$	$\ell ^2$	$\ell ^2$	$\ell$
Baseline LMEM	$\ell ^3 N$	$\ell ^3 N$	*N*	*N*	$\ell ^3 N$	$\ell ^3 N$	$\log \ell +\log N$

BTime and Bsize are generation time and size of BTs. Dtime and Dsize are generation time for the shares of the database and size of the shares. Stime is the time for Search phase. Comm. is the size of data exchanged between computing nodes. Round is the number of data exchanges

## Proposed protocols

### Problem setting and outline of our protocols

We assume that a query holder $\mathcal {A}$, a database holder $\mathcal {B}$, and two computing nodes $P_0$ and $P_1$ participate the protocol. $\mathcal {A}$ holds a query string $w$ of length $\ell$ and $\mathcal {B}$ holds a database string $T$ of length $N$. After the protocol is run, only $\mathcal {A}$ knows LPM or LMEM between $w$ and $T$. $P_0$ and $P_1$ do not obtain any information of $w$ and $T$, except for $\ell$ and $N$.

Our protocol consists of offline, DB preparation, and Search phases. In the offline phase, $\mathcal {B}$ generates BTs (correlated randomness used for multiplication) and sends them to $P_0$ and $P_1$. In the DB preparation phase, $\mathcal {B}$ creates a lookup table and distributes its shares to $P_0$ and $P_1$. In the Search phase, $\mathcal {A}$ generates shares of the query and sends them to $P_0$ and $P_1$, and $P_0$ and $P_1$ jointly compute the result without obtaining any information of the lookup table. Finally, $\mathcal {A}$ obtains the results. Figure 2 shows the schematic view of our goal and model. Note that the offline and DB preparation phases do not depend on a query string, so they can be computed in advance for multiple queries.

In section "Secret-shared recursive oblivious transfer", we propose the important building block ss-ROT that enables recursive reference to a lookup table. In section "Secure LPM", we describe how to design the lookup table based on FM-Index, and propose an efficient protocol for LPM by using the lookup table and ss-ROT. In section "Secure LMEM", we describe the additional table design for auxiliary data structures, and propose the complete protocol for LMEM. Table 2 summarizes the theoretical complexities of the three protocols. For comparison, the complexities of the baseline protocols and a previous method for LPM based on an additive homomorphic encryption [17, 19] are shown. As we mentioned in section "Introduction", the baseline protocols are designed using major techniques of secret-sharing-based protocols. The detailed algorithms are described in Appendix 3.

### Secret-shared recursive oblivious transfer

We define a problem called a secret-shared recursive oblivious transfer (ss-ROT) as follows.

#### **Definition 1**

We assume a database holder $\mathcal {B}$ and two computing nodes $P_0$ and $P_1$ participate the protocol. $\mathcal {B}$ holds a vector *V* of length $N$ and $0 \le V[i] < N$. Given the initial position $p_0$ and the depth of recursion $\ell$ $(2 \le \ell )$, the secret-shared recursive oblivious transfer protocol outputs shares of

3 $$\begin{aligned} \underbrace{V[V[\cdots V}_{\ell }[ p_0 ]\cdots ]] \end{aligned}$$

without leaking *V* to $P_0$ and $P_1$.

For simplicity, we denote the recursion of Eq. 3 by $V^{(\ell )}[p_0]$ (e.g., $V[V[p_0]]$ is denoted by $V^{(2)}[p_0]$). In our protocol, all the random values are uniformly generated from $\mathbb {Z}_{2^n}$.

*DB preparation phase* $\mathcal {B}$ generates $\ell -1$ random values $r^0,\ldots ,r^{\ell -2}$ and computes the following vectors $R^0 , \ldots , R^{\ell -1}$. Each vector $R^j$ has $N$ elements.

4 $$\begin{aligned} R^j[i] = {\left\{ \begin{array}{ll} (V[i]+r^j)_{\bmod {N}} &{} (j=0) \\ (V[(i-r^{j-1})_{\bmod {N}}]+r^j)_{\bmod {N}} &{} (1\le j\le \ell - 2) \\ (V[(i-r^{j-1})_{\bmod {N}}])_{\bmod {N}} &{} (j= \ell -1) \\ \end{array}\right. } \end{aligned}$$

$\mathcal {B}$ computes $\mathsf {Share}(R^j[i])$ and sends $[\![R^j[i]]\!]_0$ and $[\![R^j[i]]\!]_1$ to $P_0$ and $P_1$, for $i=0, \ldots , N-1$ and $j=0,\ldots ,\ell -1$.

**Fig. 3:**
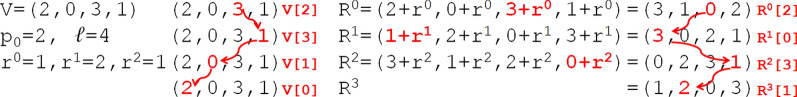
Example of a search when $V=(2,0,3,1)$, $p_0=2$, and $\ell =4$. The goal is to compute $[\![ V^{(4)}[2] \, ]\!]=[\![2]\!]$. Here we assume $\mathcal {B}$ generates $r^0=1, r^1=2, r^2=1$. In Step 1 of Search phase, $P_0$ and $P_1$ jointly compute $\mathsf {Reconst}([\![R^0[2]\,]\!]_0, [\![R^0[2]\, ]\!]_1)$ to obtain $R^0[2]=0$. ($R^0[2]$ is randomized by $r^0$, so any element of *V* is leaked.) In a similar way, $P_0$ and $P_1$ compute $R^1[0] = 3$ and $R^2[3] = 1$. In Step 2, $P_0$ and $P_1$ output $[\![R^3[1]\, ]\!]_0$ and $[\![R^3[1]\, ]\!]_1$ respectively. Since $R^0[2]=V[2]+r^0$, $R^1[V[2]+r^0] = V[V[2]+r^0-r^0]+r^1$, $R^2[V[V[2]]+r^1] = V[V[V[2]] +r^1-r^1 ]+r^2$, and $R^3[V[V[V[2]]]+r^2] = V[V[V[V[2]]] +r^2-r^2]$, ss-ROT successfully computes $[\![ V^{(4)}[2] \, ]\!]$

*Search phase* The Search phase consists of two steps and is described in Lines 2–5 of Protocol 1. The input is the initial position $p_0$ and shares of *R*. The output is $[\![V^{(\ell )}[p_0] ]\!]$. An example of a search is illustrated in Fig. 3.

#### Security intuition

In the DB preparation phase of ss-ROT, $\mathcal {B}$ does not disclose any private values, and $P_0$ and $P_1$ receive the shares. In the Search phase, all the messages exchanged between $P_0$ and $P_1$ are shares except for the result of $\mathsf {Reconst}$ in Step 1. In the *j*-th step of the loop in Step 1, $p_{j+1} = R^j[p_{j}] = (V^{(j+1)}[p_0]+r^{j})_{\bmod {N}}$ is reconstructed. Since the reconstructed value is randomized by $r^{j}$, no information is leaked. Note that for each vector $R^j$, all the elements $R^j[0], \ldots , R^j[N-1]$ are randomized by the same value $r^{j}$, but only one of them is reconstructed, and different random numbers $r^{0}, \ldots , r^{\ell -1}$ are used for $R^0, \ldots , R^{\ell -1}$. In Step 2, $P_0$ and $P_1$ output a result, and no information other than the result is leaked.

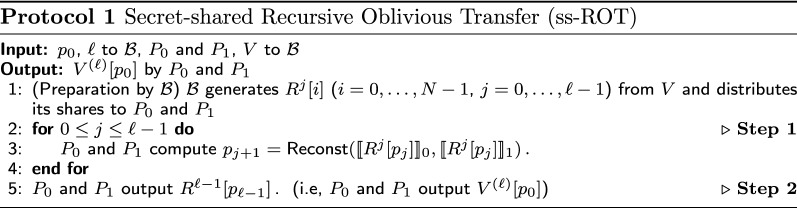



#### Security

##### **Theorem 1**

*ss-ROT  is correct and secure in the semi-honest model.*

##### *Proof*

Correctness and security of ss-ROT protocol are proved as follows.

*Correctness.* We assume the following equation.

5 $$\begin{aligned} p_{i} = (V^{(i)}[p_0]+r^{i-1})_{\bmod {N}} \end{aligned}$$

In Step1, for $j=0$, the protocol computes $p_{1}$ by reconstructing $R^0[p_0]$. From the definition of $R^j[i]$ in Eq. 4,

6 $$\begin{aligned} p_{1} = R^0[p_0] = (V^{(1)}[p_0]+r^0)_{\bmod {N}}. \end{aligned}$$

For $j=k$, the protocol computes $p_{k+1}$ by reconstructing $R^{k}[p_k]$. From the definition of $R^j[i]$ in Eq. 4 and the assumption of Eq. 5,

7 $$\begin{aligned} p_{k+1} = R^{k}[p_k]= & {} (V[\, (p_k - r^{k-1})_{\bmod {N}} \,]+r^k)_{\bmod {N}} \nonumber \\= & {} (V[\, V^{(k)}[p_0] \,] +r^k)_{\bmod {N}} \nonumber \\= & {} (V^{(k+1)}[p_0]+r^k)_{\bmod {N}}. \end{aligned}$$

Eq. 5 holds for $i=1$ by Eq. 6. It also holds for $i=k+1$ under the assumption that Eq. 5 holds for $i=k$. Therefore by induction, Eq. 5 holds for $i=1,\ldots , \ell -1$.

In Step 2, $P_0$ and $P_1$ output $[\![R^{\ell -1}[p_{\ell -1}]]\!]$. Since Eq. 5 holds for $i=\ell -1$,

$$\begin{aligned} R^{\ell -1}[p_{\ell -1}] = (V[( p_{\ell -1} -r^{\ell -2})_{\bmod {N}}])_{\bmod {N}} \end{aligned}$$

is transformed into $(V^{(\ell )}[p_0])_{\bmod {N}}$ by plugging in $p_{\ell - 1} = V^{(\ell -1)}[p_0]+r^{\ell -2}$. Therefore the final output of ss-ROT becomes $(V^{(\ell )}[p_0])_{\bmod {N}}$. The above argument completes the proof of correctness of Theorem 1.

*Security.* Since the roles of $P_0$ and $P_1$ are symmetric, it is sufficient to consider the case when $P_0$ is corrupted. The input to $P_0$ is $p_0$ and $\ell$, and output of $P_0$ is $V^{(\ell )}[p_0]$. The function achieved by Protocol 1 is deterministic and the protocol is correct. Therefore, to ensure the security of Protocol 1, we need to prove existence of a probabilistic polynomial-time simulator ${\mathcal {S}}$ such that

8 $$\begin{aligned} \{(\mathcal {S}(p_0, \ell , V^{(\ell )}[p_0]), V^{(\ell )}[p_0])\} \equiv \{(X, V^{(\ell )}[p_0])\}, \end{aligned}$$

where *X* is $P_0$’s view. *X* consists of:

- $[\![ R^j[i] ]\!]_0$ for $i=0,\ldots ,N-1$ and $j=0,\ldots ,\ell -1$ (a message from $\mathcal {B}$)
- $[\![ R^j[p_j] ]\!]_1$ (*j*-th message from $P_1$) for $j=0,\ldots ,\ell -1$
- $p_j$ (*j*-th value obtained by $\mathsf {Reconst}([\![R^j[p_{j}]]\!]_0, [\![R^j[p_{j}]]\!]_1)$ in **Step1**) for $j=1,\ldots ,\ell -1$.

All the messages from $\mathcal {B}$ and $P_1$ are uniformly at random in $\mathbb {Z}_{2^n}$, as they are generated by $\mathsf {Share}$. $p_j+1 = \mathsf {Reconst}([\![R^j[p_{j}]]\!]_0, [\![R^j[p_{j}]]\!]_1)$ holds for $j=0,\ldots ,\ell -2$, and $V^{(\ell )}[p_0] = \mathsf {Reconst}([\![R^{\ell -1}[p_{\ell -1}]]\!]_0, [\![R^{\ell -1}[p_{\ell -1}]]\!]_1)$ holds. $p_1=R^{0}[p_{0}],\; p_2=R^{1}[p_{1}], \ldots , p_{\ell -1}=R^{\ell -2}[p_{\ell -2}]$ are uniformly at random in $\mathbb {Z}_{N}$ from the definition of Eq. 4.

Let us denote a random number *u* chosen from a set ${\mathcal {U}}$ uniformly at random by $u{\mathop {\in }\limits ^{R}} {\mathcal {U}}$. We construct ${\mathcal {S}}$ as described in Protocol 2. The output of ${\mathcal {S}}$ is $\tilde{R_0} \in \mathbb {Z}_{2^n}^{\ell \times N}$, $\tilde{R_1} \in \mathbb {Z}_{2^n}^{\ell }$, and $\tilde{p_1},\ldots ,\tilde{p}_{\ell -1}$. In Line 6 and Line 9, $\tilde{p_1},\ldots ,\tilde{p}_{\ell -1}$ are generated such that they are uniformly at random in $\mathbb {Z}_{N}$. In Line 7, $\tilde{R_0}^{j}[{p_0}]$ and $\tilde{R_1}[0]$ are generated by $\mathsf {Share}$ such that they are shares of $\tilde{p}_1$ and uniformly at random in $\mathbb {Z}_{2^n}$. In Line 10, $\tilde{R_0}^{j}[{\tilde{p}_j}]$ and $\tilde{R_1}[j]$ are generated by $\mathsf {Share}$ such that they are shares of $\tilde{p}_{j+1}$ and uniformly at random in $\mathbb {Z}_{2^n}$ for $j=1,\ldots ,\ell -2$. In Line 12, $\tilde{R_0}^{j}[\tilde{p}_{\ell -1}]$ and $\tilde{R_1}[\ell -1]$ are generated by $\mathsf {Share}$ such that they are shares of $V^{(\ell )}[p_0]$ and uniformly at random in $\mathbb {Z}_{2^n}$. All the elements of $\tilde{R_0}$ except for $\tilde{R_0}^{0}[p_0]$ and $\tilde{R_0}^{j}[\tilde{p}_j]$ ($j=1,\ldots ,\ell -1$) are uniformly at random in $\mathbb {Z}_{2^n}$ by Line 3. Therefore, Eq. 8 holds. By the above discussion, we find our ss-ROT satisfies security in the semi-honest model. $\square$


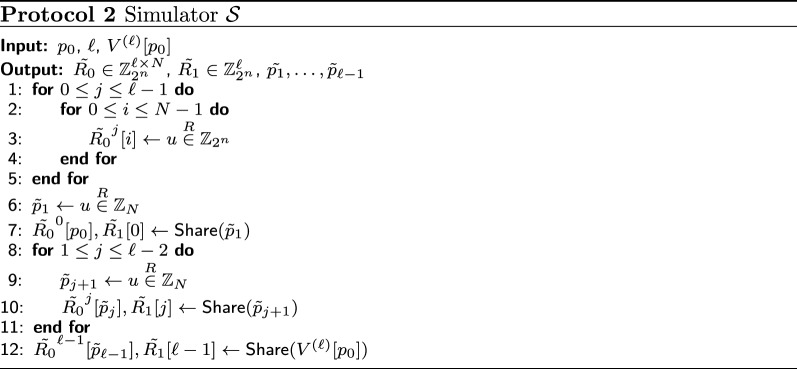


#### Complexities

In the DB preparation phase, $\mathcal {B}$ generates shares of *V* of length $N$ for $\ell$ times. Therefore, time and communication complexities are $O(\ell N)$. For the Search phase, $\mathsf {Reconst}$ is computed $\ell$ times in Step 1. Since the time, communication, and round complexities of $\mathsf {Reconst}$ are *O*(1), those of the Search phase become $O(\ell )$.

### Secure LPM

*Construction of lookup table* The goal is to find LPM securely. To apply FM-Index for a prefix search, the reverse string of $T$ (i.e., $\hat{T}$) is used. The backward search of FM-Index is formulated by Eq. 1. If we precompute $\mathsf {LF}_c(i, \hat{T})$ for $i=0, \ldots , N$ and $c \in \{$A,T,G,C$\}$, and store them in a lookup table that consists of four vectors: $V_\mathrm{A}$, $V_\mathrm{C}$, $V_\mathrm{G}$, and $V_\mathrm{T}$ such that $V_c[i] = \mathsf {LF}_c(i, \hat{T})$, Eq. 1 is replaced by the following table lookup

9 $$\begin{aligned} f_{k+1}=V_{w[k]}[f_k],\qquad g_{k+1}=V_{w[k]}[g_k]. \end{aligned}$$

I.e., starting with the initial interval $$[f_0 = 0, g_0 = N)$$, we can compute the match by recursively referring to the lookup table while $$f<g$$.

*Protocol overview* The key idea of Secure LPM is to refer to *V* by ss-ROT, i.e., $P_0$ and $P_1$ jointly refer to *V* $\ell$ times in a recursive manner. To achieve backward search, $P_0$ and $P_1$ need to select $V_x[\cdot ]$ for each reference, where *x* is a query letter to be searched with. This is achieved by expressing the query letter by unary code (Eq. 11 ) and computing the inner product of Eq. 11 and $(V_\mathrm{A}[\cdot ], V_\mathrm{C}[\cdot ], V_\mathrm{G}[\cdot ], V_\mathrm{T}[\cdot ])$. To find LPM, $P_0$ and $P_1$ need to check $f=g$ for each reference. We use the subprotocol $\mathsf {Equality}$ to check it securely. Since *V* is randomized with different numbers for searching *f* and *g*, the difference of the random numbers is precomputed and removed securely upon the equality check. $\mathcal {A}$ receives only the result of each equality check to know LPM. For example, LPM is the prefix of length $i-1$ when $f=g$ for the *i*-th reference. If $f \ne g$ for all references, LPM is the entire query.

*DB preparation phase*$\mathcal {B}$ creates a lookup table and generates the following $4\ell$ vectors in a similar manner to ss-ROT. For simplicity, we denote the length of $V_c$ by $N'=N+1$.

10 $$\begin{aligned} R_{c,f}^j[i]= {\left\{ \begin{array}{ll} (V_c[i]+r_f^j)_{\bmod {N'}} &{} (j=0)\\ (V_c[(i-r_{f}^{j-1})_{\bmod {N'}}]+r_f^j)_{\bmod {N'}} &{} (1\le j <\ell ) \end{array}\right. } \end{aligned}$$

$R_{c,f}^j[i]$ is used for computing the lower bound *f* of the interval [*f*, *g*). We also generate $R_{c,g}^j[i]$ for the upper bound *g*. *R* consists of $8\ell$ vectors, each of length $N'$. Since the longest match is found when $f=g$, $\mathcal {B}$ also generates a vector $r'[j]=(r_f^j-r_g^j)_{\bmod {N'}}$ that is used for equality check of *f* and *g*. Then, $\mathcal {B}$ sends shares of $R_{c,f}^j[i]$, $R_{c,g}^j[i]$, and $r'[j]$ to $P_0$ and $P_1$.

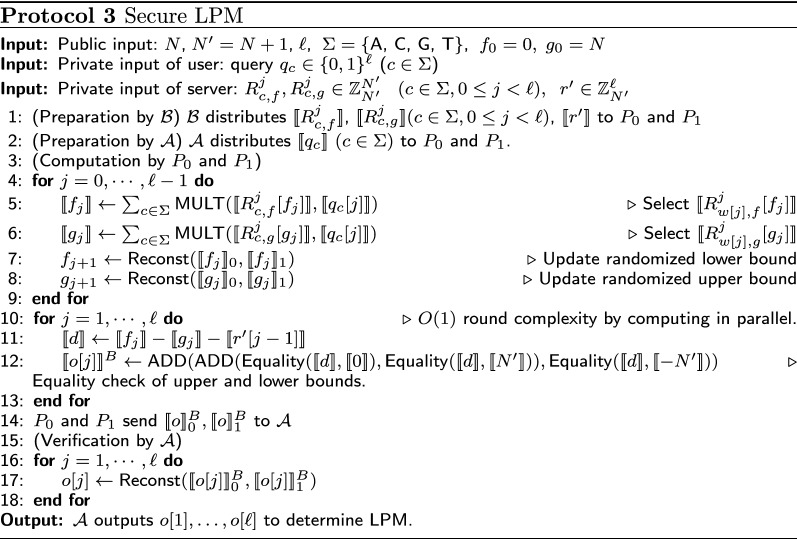



*Search phase* Protocol 3 describes the algorithm in detail. $\mathcal {A}$ generates four vectors ${q}_\mathrm{A}$, ${q}_\mathrm{C}$, ${q}_\mathrm{G}$, ${q}_\mathrm{T}$, each of length $\ell$, as follows.

11 $$\begin{aligned} q_c[j] = {\left\{ \begin{array}{ll} 1 &{} (c=w[j])\\ 0 &{} (c\ne w[j]) \end{array}\right. } \end{aligned}$$

**Fig. 4:**
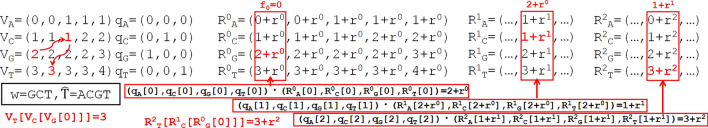
Example of a secure table lookup when $w$ = GCT and $\hat{T}$ = ACGT. Only the lookup for a lower bound is shown. For simplicity, $R^j_{c,f}$ and $r^j_f$ are denoted by $R^j_{c}$ and $r^j$. $\mathsf {LF}_{w[i]}(f_i,\hat{T})$ ($i=0,1,2$) is computed by $V_{\mathrm{G}}[0], V_{\mathrm{C}}[2]$, and $V_{\mathrm{T}}[1]$. *V* is referenced securely by using *R*. $R^0_{\mathrm{G}}[0]$ is computed by $\sum _{c\in \Sigma } q_c[0] \cdot R_c[0]$. $R^1_{\mathrm{C}}[2+r^0]$ is computed by $\sum _{c\in \Sigma } q_c[1] \cdot R_c[2+r^0]$. $R^2_{\mathrm{T}}[1+r^1]$ is computed by $\sum _{c\in \Sigma } q_c[2] \cdot R_c[1+r^1]$

For each *j*, $(q_\mathrm{A}[j], q_\mathrm{C}[j], q_\mathrm{G}[j], q_\mathrm{T}[j])$ encodes $w[j]$ (e.g., $(q_\mathrm{A}[j], q_\mathrm{C}[j], q_\mathrm{G}[j], q_\mathrm{T}[j])=(1,0,0,0)$ if $w[j]=\mathrm{A}$). The aim of the encode is to compute $[\![ R_x[j] ]\!] = [\![ \sum _{c\in \Sigma } q_c[j] \cdot R_c[j] ]\!]$ when $w[j]=x$. Figure 4 illustrates an example of the table lookup.

$$\mathcal {A}$$ generates shares of $${q}_\mathrm{A}$$, $${q}_\mathrm{C}$$, $${q}_\mathrm{G}$$, $${q}_\mathrm{T}$$ and distributes them to $$P_0$$ and $$P_1$$. $$P_0$$ and $$P_1$$ compute $$\mathsf {LF}_{w[j]}(f',\hat{T}) + r^j_f$$ and $$\mathsf {LF}_{w[j]}(g',\hat{T}) + r^j_g$$ in Lines 5–8 without leaking $$f'$$ and $$g'$$, where $$[f', g')$$ corresponds to the match of *w*[0, *j*] and $$\hat{T}$$. In Lines 10–13, the equality of $$f'$$ and $$g'$$ is examined for all rounds. Note that different values $$r^{j-1}_f$$ and $$r^{j-1}_g$$ are used for $$f_j = (f'-r^{j-1}_f)_{\bmod {N'}}$$ and $$g_j = (g'-r^{j-1}_g)_{\bmod {N'}}$$ in order to conceal $$f'$$ and $$g'$$. Since $$f'$$, $$g'$$, $$r^{j-1}_f$$, $$r^{j-1}_g, r'[j-1]\in \{0,\ldots ,N'-1\}$$, it is sufficient to check if $$f_j - g_j - r'[j-1]$$ is equal to either one of $$-N', 0,$$ and $$N'$$. In Lines 16–18, $$\mathcal {A}$$ receives all the results of equality checks (i.e., $$[\![o[1]]\!]^B ,\ldots ,[\![o[\ell ]]\!]^B$$) from $$P_0$$ and $$P_1$$, and knows LPM by reconstructing them. For example, if $$w=$$GCT and $$o=(0,0,1)$$, $$\mathcal {A}$$ knows that LPM is GC.

#### Security

##### **Theorem 2**

*Protocol 3 is correct and secure in the semi-honest model.*

##### *Proof*

Correctness and security of Protocol 3 are proved as follows.

*Correctness.* The lookup table *V* simply stores all possible outputs of $\mathsf {LF}$. Therefore, backward search (Eq. 1) is equivalent to Eq. 9. For the case of querying *w*, $V_{w[k-1]}[\cdots V_{w[0]} [ p_0 ]\cdots ]$ becomes lower bound *f* (for $p_0=0$) or upper bound *g* (for $p_0=N$) of the interval that corresponds to the prefix match of length *k*. In Line 5 of Protocol 3, $[\![ R_{\mathrm{A},f}^k[f_{k}]\times q_\mathrm{A}[k] + R_{\mathrm{C},f}^k[f_{k}]\times q_\mathrm{C}[k] + R_{\mathrm{G},f}^k[f_{k}]\times q_\mathrm{G}[k] + R_{\mathrm{T},f}^k[f_{k}]\times q_\mathrm{T}[k] ]\!]$ is computed. Since $q_{w[j]}[j]=1$ and $q_{c}[j]=0$ ($c \ne w[j]$), it is equivalent to $[\![ R_{w[k],f}^k[f_{k}] ]\!]$. Line 6 computes $[\![ R_{w[k],g}^j[g_{k}] ]\!]$ in the same manner. Each vector $R_{c, f}^j$ in Eq. 10 is generated in the same manner as $R^j$ in Eq. 4. Since Eq. 10 uses the common random values $r_f^j$ and $r_f^{j-1}$ for $R_{\mathrm{A}, f}^j$, $R_{\mathrm{C}, f}^j$, $R_{\mathrm{G}, f}^j$, $R_{\mathrm{T}, f}^j$, we can recursively reference $V_c$ ($c \in \{$A, C, G, T$\}$), which is obvious from the correctness of ss-ROT. Therefore, the recursion by Line 5 and Line 7 can compute $(V_{w[k-1]}[\cdots V_{w[0]} [ f_0 ]\cdots ] + r_f^{k-1})_{\bmod {N'}}$, and the recursion by Line 6 and Line 8 can also compute $( V_{w[k-1]}[\cdots V_{w[0]} [ g_0 ]\cdots ] + r_g^{k-1})_{\bmod {N'}}$.

The longest match is found when the interval width becomes 0. Since $f_k = ( V_{{w}[k-1]}[\cdots V_{w[0]} [ f_0 ]\cdots ] + r^{k-1}_f)_{\bmod {N'}}$ and $g_k = ( V_{{w}[k-1]}[\cdots V_{w[0]} [ g_0 ]\cdots ] + r^{k-1}_g) _{\bmod {N'}}$ are randomized, Line 11 computes $f_k-g_k-(r'[k-1] = (r^{k-1}_f - r^{k-1}_g)_{\bmod {N'}})$ to obtain the correct interval width. When the width is 0, *d* becomes either one of 0, $N'$ and $-N'$. Therefore, Line 12 computes the equality *d* and 0, $N'$ and $-N'$ respectively. By reconstructing all the results in Lines 16–18, $\mathcal {A}$ knows the round, in which the interval width becomes 0; i.e., he/she knows LPM. The above argument completes the proof of correctness of Theorem 2.

*Security* We only show a sketch of the proof. For Lines 1–2 of Protocol 3, $\mathcal {A}$ and $\mathcal {B}$ do not disclose any private values, and $P_0$ and $P_1$ receive the shares. For Lines 3–14, it is guaranteed by the subprotocols $\textsf {ADD}$, $\textsf {MULT}$, and $\mathsf {Equality}$ that all the messages exchanged between $P_0$ and $P_1$ are shares except for the output of $\mathsf {Reconst}$ in Lines 7–8. (see section "Secure computation based on secret sharing" for details of the subprotocols.) In Lines 7–8, reconstructed values are $R_{{w}[j],f}^k[f_{j}]$ and $R_{{w}[j],g}^k[g_{j}]$. Since the values are $(V_{{w}[j]}[f_j] + r_f^{j})_{\bmod {N'}}$ and $(V_{{w}[j]}[g_j] + r_g^{j})_{\bmod {N'}}$ according to Eq. 10, it is obvious that *V* is randomized for all rounds $j=0,\ldots , \ell -1$, and no information is leaked. For Lines 14–17, only the output of $\mathsf {Equality}$ at Line 11 is reconstructed. The reconstructed values are either 1 or 0 according to $\mathsf {Equality}$, and no information other than the result is leaked. $\square$

$\mathcal {A}$ may reveal $T$ by making many queries. Such a problem is called output privacy. Although output privacy is outside of the scope of this paper, we should mention here that $\mathcal {A}$ needs to make an unrealistically large number of queries for obtaining $T$ by such a brute-force attack, considering that $N$ is very long.

#### Complexities

The DB preparation phase generates shares of $R^j_{c, f}$ and $R^j_{c, g}$ ($c \in \Sigma$ and $0 \le j < \ell$); i.e., $8\times \ell$ vectors of length $N'$. Therefore, the time and communication complexities are $O(\ell N)$. For the Search phase, $\textsf {MULT}$ and $\mathsf {Reconst}$ are computed twice in Lines 4–9 for $\ell$ rounds and $\mathsf {Equality}$ is computed once in Lines 10–13 for $\ell$ rounds. Note that $\mathsf {Equality}$ is computed in parallel, and the number of round can be reduced to a constant number. Each time, the communication and round complexities of these subprotocols are *O*(1), so those of the Search phase become $O(\ell )$.

### Secure LMEM

*Construction of lookup table* As described in section "Index structure for string search", we can find a parent interval by a reference to $\mathsf {LCP}$, $\mathsf {PSV}$, and $\mathsf {NSV}$. Therefore, in addition to $V_c$ defined in section "Secure LPM", we prepare lookup tables that simply store all the outputs of them; i.e., $V_{lcp}[i]=\mathsf {LCP}[i]$, $V_{psv}[i] = \mathsf {PSV}[i]$, and $V_{nsv}[i] = \mathsf {NSV}[i]$.

*DB preparation phase* $\mathcal {B}$ generates randomized vectors $R_{c,f}$, $R_{c,g}$ and $r'[j]=(r_f^j-r_g^j)_{\bmod {N'}}$ using the same algorithm in section "Secure LPM" for length $2\ell$. As shown in Eq. 2, $V_{lcp}$ is referred by the upper and lower bounds of [*f*, *g*). Therefore, $\mathcal {B}$ generates following circular permutations of $V_{lcp}$ such that $W_{l,f}$ and $R_{c,f}$, and $W_{l,g}$ and $R_{c,g}$, are permutated by the same random values, respectively. I.e.,

$$\begin{aligned} W_{l,x}^j[i]= {\left\{ \begin{array}{ll} V_{lcp}[i] &{} (j=0)\\ V_{lcp}[(i-r_{x}^{j-1})_{\bmod {N}}] &{} (1\le j <2\ell ) , \end{array}\right. } \end{aligned}$$

where *x* is either *f* or *g*. $V_{psv}$ is referred by both *f* and *g*, and is plugged in to *f*. Therefore, $\mathcal {B}$ generates $W_{p,f}^j$ and $W_{p,g}^j$ such that both of them are randomized by $r_f^j$, and $W_{p,f}^j$ is permutated by $r_f^{j-1}$ and $W_{p,g}^j$ is permutated by $r_g^{j-1}$ as follows.

$$\begin{aligned}&W_{p,f}^j[i]= {\left\{ \begin{array}{ll} (V_{psv}[i]+r_f^j)_{\bmod {N}} &{} (j=0)\\ (V_{psv}[(i-r_{f}^{j-1})_{\bmod {N}}]+r_f^j)_{\bmod {N}} &{} (1\le j<2\ell ) \end{array}\right. } \\&W_{p,g}^j[i]= {\left\{ \begin{array}{ll} (V_{psv}[i]+r_g^j)_{\bmod {N}} &{} (j=0)\\ (V_{psv}[(i-r_{g}^{j-1})_{\bmod {N}}]+r_f^j)_{\bmod {N}} &{} (1\le j <2\ell ) \end{array}\right. } \end{aligned}$$

Similarly, $V_{nsv}$ is referred by both *f* and *g*, and is plugged in to *g*. Therefore, $\mathcal {B}$ generates $W_{n,f}^j[i]$ and $W_{n,g}^j[i]$ as follows.

$$\begin{aligned}&W_{n,f}^j[i]= {\left\{ \begin{array}{ll} (V_{nsv}[i]+r_f^j)_{\bmod {N}} &{} (j=0)\\ (V_{nsv}[(i-r_{f}^{j-1})_{\bmod {N}}]+r_g^j)_{\bmod {N}} &{} (1\le j<2\ell ) \nonumber \end{array}\right. } \\&W_{n,g}^j[i]= {\left\{ \begin{array}{ll} (V_{nsv}[i]+r_g^j)_{\bmod {N}} &{} (j=0)\\ (V_{nsv}[(i-r_{g}^{j-1})_{\bmod {N}}]+r_g^j)_{\bmod {N}} &{} (1\le j <2\ell ) \end{array}\right. } \end{aligned}$$

$\mathcal {B}$ distributes shares of $R_{c,f}$, $R_{c,g}$, $r'$, $W_{l,f}$, $W_{l,g}$, $W_{p,f}$, $W_{p,g}$, $W_{n,f}$, and $W_{n,g}$ to $P_0$ and $P_1$.

*Search phase* Protocol 4 describes the algorithm in detail. $$\mathcal {A}$$ generates query vectors $${q}_\mathrm{A}$$, $${q}_\mathrm{C}$$, $${q}_\mathrm{G}$$, $${q}_\mathrm{T}$$ by Eq. 11 and distributes shares of the vectors to $$P_0$$ and $$P_1$$. In Line 6 of Protocol 4, $$[{\hat{f}}, {\hat{g}})$$ is computed by the reference to *R* (i.e., a search based on a backward search) similarly to Lines 5–6 of Protocol 3. In Line 11, $$[f_{ex}, g_{ex})$$ is computed by the reference to *W* (i.e., a search based on $$\mathsf {LCP}$$, $$\mathsf {PSV}$$ and $$\mathsf {NSV}$$). In Line 13, the interval is updated by either $$[{\hat{f}}, {\hat{g}})$$ or $$[f_{ex}, g_{ex})$$ based on the result of $$f' = g'$$ in Lines 7–9, where $$[f', g')$$ corresponds to the interval that corresponds to a substring match.

In each round, we need to know a query letter to be searched with, so we need to maintain the right end position of the match in the query. The position moves toward the right while the match is extended, but remains the same when the interval is updated based on $\mathsf {PSV}$ and $\mathsf {NSV}$. To memorize the position, we prepare shares of a unit bit vector *u* of length $\ell$, in which the position *t* is memorized as $u[t] = 1$ and $u[i \ne t] = 0$. In Lines 20–23, *u* remains the same if the interval is updated based on $\mathsf {PSV}$ and $\mathsf {NSV}$, and $u = (0, u[0], u[1], \ldots , u[\ell -2])$ otherwise. When the search is finished (e.g., the right end of a match exceeds the right end of the query) $u = (0, \ldots , 0)$. Therefore in Lines 25–28, $x=1$ while the right end of a match dose not exceed the right end of the query and $x=1$ after finishing the search. In Lines 29–31, the inner product of $q_c$ ($c \in \Sigma$) and *u* becomes the encode of *w*[*t*] that is used for the next round.

We also maintain the left end position of the match. While the match is extended, the position remains the same and it moves toward the right when the interval is updated by $$[f_{ex}, g_{ex})$$. The new left end position can be computed by $$p+m-c$$ where *p* is the current position, *m* is the length of the current match, and *c* is the lcp-value of $$[f_{ex}, g_{ex})$$ (i.e., the longest common prefix length of suffixes contained in $$[f_{ex}, g_{ex})$$). The position is computed in Line 33. The match length is incremented by 1 for each extension while the right end of the match does not exceed the query length. When the interval is updated by $$[f_{ex}, g_{ex})$$, the match length is reduced to the lcp-value of $$[f_{ex}, g_{ex})$$, which is computed by $$\max (\mathsf {LCP}[f], \mathsf {LCP}[g])$$. The match length is computed in Line 32. In Line 35, the longest match length and the corresponding left end position are updated. After all the positions in the query have been examined, LMEM and its left end position are sent to $$\mathcal {A}$$ in Line 37.

#### Security

##### **Theorem 3**

*Protocol 4 is correct and secure in the semi-honest model.*

##### *Proof*

Correctness and security of Protocol 4 are proved as follows. *Correctness.* *V*, *R*, $$r'$$ and *q* are generated by the same algorithm used in Protocol 3. Therefore, Line 6 is equivalent to a backward search, and *e*1 is the result of the equality check of 0 and the width of the obtained interval in Lines 7-8. The lookup tables $$V_{lcp}$$, $$V_{psv}$$, and $$V_{nsv}$$ store all the outputs of $$\mathsf {LCP}$$, $$\mathsf {PSV}$$ and $$\mathsf {NSV}$$, and $$W_l$$, $$W_p$$, and $$W_n$$ are generated based on $$V_{lcp}$$, $$V_{psv}$$, and $$V_{nsv}$$, respectively. Since $$W_{l, f}^j$$ and $$W_{l, g}^j$$ are circular permutations of $$V_{lcp}$$ by the same random values $$r_f^{j-1}$$ and $$r_g^{j-1}$$ that are used for generating $$R_{c, f}$$ and $$R_{c, g}$$ $$(c\in \Sigma )$$ respectively, Line 8 can compute $$\mathsf {LCP}[g_j] \le \mathsf {LCP}[f_j]$$ and *e*2 holds the result. By using $$\mathsf {Choose}$$ and *e*2, either $$[W_{p, f}^j[f_j], W_{n, f}^j[f_j])$$ or $$[W_{p, g}^j[g_j], W_{n, g}^j[g_j])$$ is selected. $$W_{p, f}^j$$ and $$W_{p, g}^j$$ are permutated by $$r_f^{j-1}$$ and $$r_g^{j-1}$$, but are randomized by the identical random value $$r_f^{j}$$. Similarly, $$W_{n, f}^j$$ and $$W_{n, g}^j$$ are permutated by $$r_f^{j-1}$$ and $$r_g^{j-1}$$, but are randomized by $$r_g^{j}$$. Since $$W_{p, f}[f_j]$$ and $$W_{n, g}^j[g_j]$$ are generated in the same manner as $$R_{c,f}$$ and $$R_{c,g}$$, it is obvious that the reference by them is correct. The reference by $$W_{n, f}^j[f_j]$$ is transformed into

12 $$\begin{aligned} X_g^{j+1}[ W_{n, f}^j[ f_j ] ] = & {} V_{x}[ W_{n, f}^j [ f_j ] - r_g^{j} ] + r_g^{j+1} \nonumber \\= & {} V_{x}[ V_{nsv}[ f_j - r_f^{j-1} ] + r_g^j - r_g^{j} ] + r_g^{j+1} \nonumber \\= & {} V_{x}[ \; V_{nsv}[ f_j - r_f^{j-1}] \; ] + r_g^{j+1} \end{aligned}$$

and the reference by $W_{p, f}^j[g_j]$ is transformed into

13 $$\begin{aligned} X_f^{j+1}[ W_{p, g}^j[ g_j ] ] = & {} V_{x}[ W_{p, g}^j [ g_j ] - r_f^{j} ] + r_f^{j+1} \nonumber \\= & {} V_{x}[ V_{psv}[ g_j - r_g^{j-1} ] + r_f^j - r_f^{j} ] + r_f^{j+1} \nonumber \\= & {} V_{x}[ \; V_{psv}[ g_j - r_g^{j-1}] \; ] + r_f^{j+1} \end{aligned}$$

where $$X^{j+1}$$ is any one of $$R^{j+1}_c$$, $$W^{j+1}_{p}$$ and $$W^{j+1}_{n}$$, and $$V_{x}$$ is the corresponding lookup table; i.e., either one of $$V_c$$, $$V_{psv}$$ and $$V_{nsv}$$. Note that $$V_{x}$$ could be a different table for each $$j+1$$, but we abuse the same notation for simplicity of notation. Since $$f_j$$ and $$g_j$$ are described in the form of $$V_x^{(j)}[p_0] + r_f^{j-1}$$ and $$V_x^{(j)}[p'_0] + r_g^{j-1}$$ based on Eq. 5, Eq. 12 and Eq. 13 are transformed into $$V_x^{(j+2)}[p_0] + r_g^{j+1}$$ and $$V_x^{(j+2)}[p'_0] + r_f^{j+1}$$, which also satisfy the recursion form of Eq. 5. Thus, the intervals $$[W_{p, f}^j[f_j], W_{n, f}^j[f_j])$$ and $$[W_{p, g}^j[g_j], W_{n, g}^j[g_j])$$ are correct intervals and Line 11 is equivalent to computing Eq. 2.

Lines 16–23, *u* remains the same if $e1=0$ and $u = (0, u[0], u[1], \ldots , u[\ell -2])$ otherwise. Therefore Lines 29–31 can choose the letter to be searched with. The match length and the start position are obtained based on *e*1 in Lines 32–33, and the longest value and the corresponding position are selected in Lines 34–35. The shares of the length and start position of LMEM are sent to $\mathcal {A}$, and $\mathcal {A}$ reconstructs them. Then, Protocol 4 outputs them. The above argument completes the proof of correctness of Theorem 3.

*Security.* We only show a sketch of the proof. For Lines 1–2 of Protocol 4, $\mathcal {A}$ and $\mathcal {B}$ do not disclose any private values, and $P_0$ and $P_1$ receive the shares. For Lines 3–37, it is guaranteed by the subprotocols $\textsf {ADD}$, $\textsf {MULT}$, $\mathsf {Equality}$, and $\mathsf {Choose}$ that all the messages exchanged between $P_0$ and $P_1$ are shares except for the output of $\mathsf {Reconst}$ in Line 14. (see section "Secure computation based on secret sharing" for details of the subprotocols.) In Line 14, the reconstructed values are $f_{i+1} = V_x^{(j+1)}[p_0] + r_f^{j}$ and $g_{j+1}=V_x^{(j+1)}[p_0] + r_g^{j}$, according to Eq. 5, Eq. 12, and Eq. 13. Since $f_{j+1}$ and $g_{j+1}$ are randomized by $r_f^{j}$ and $r_g^{j}$, respectively, for all rounds $j=0,\ldots , 2\ell -1$, no information is leaked. In Line 38, $\mathcal {A}$ reconstructs only the search result (the length and start position of LMEM). $\square$

#### Complexities

The DB preparation phase generates shares of $R^j_{c, f}$ and $R^j_{c, g}$ ($c \in \Sigma$, $0 \le j < \ell$) and $W^j_{x,f}$ and $W^j_{x,g}$ ($x \in \{{l}, p, n\}$ and $0 \le j < \ell$); $14\times \ell$ vectors of length $N+1$. Therefore, the time and communication complexities are $O(\ell N)$. For the Search phase, $\textsf {MULT}$ is computed $\ell$ times in parallel in Lines 17–18. (These are not dependent on each other.) In Line 30, $\textsf {MULT}$ is computed $\ell$ times in parallel, and Line 30 is computed in parallel four times in Lines 29–31. Lines 17–18 and Lines 29–31 are repeated for $2\ell -1$ rounds. Other subprotocols are also computed for $2\ell -1$ rounds. The time, communication, and round complexities are *O*(1) for $\textsf {MULT}$, and independent computation of $\textsf {MULT}$ for $\ell$ times does not increase the round complexity. The time, communication and round complexities are *O*(1) for the other subprotocols used in Protocol 4. Therefore, the complexities of the Search phase are $O(\ell ^2)$ for time and communication, and $O(\ell )$ for the number of rounds. The time complexity of the standard (i.e., non-privacy-preserving) LMEM is $O(\ell )$ while that of Secure LMEM is $O(\ell ^2)$. The increase in time complexity is caused by the computation for maintaining match position securely.

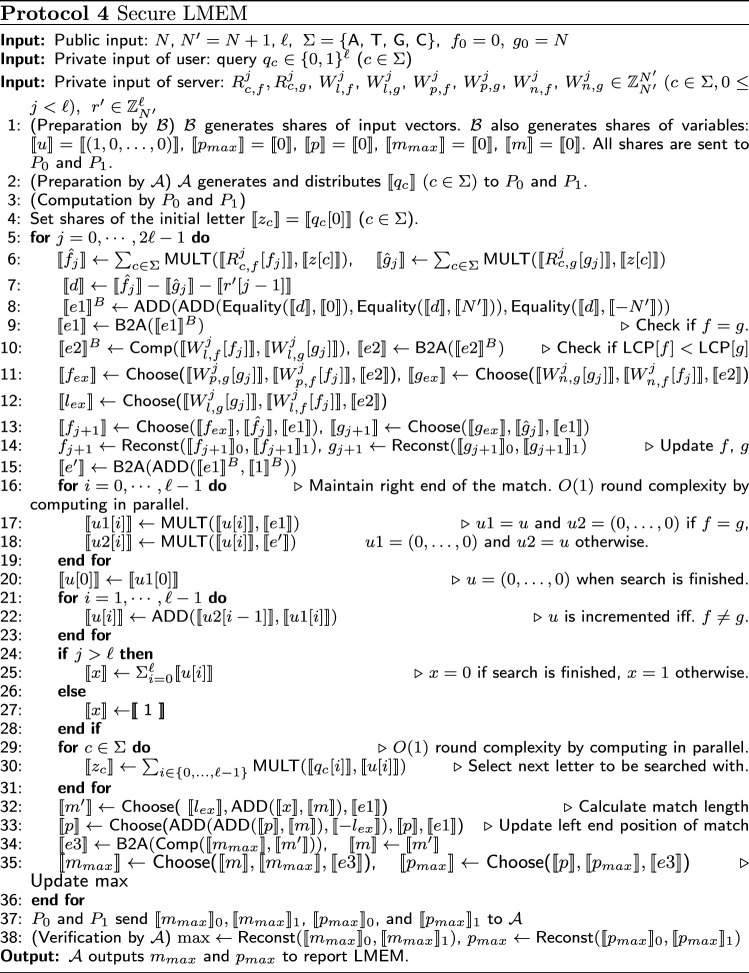



## Reducing size of shares in DB preparation phase

The protocols based on ss-ROT are quite efficient in Search phase, however, they require large data transfer from $\mathcal {B}$ to the computing nodes in DB preparation phase when the number of queries and the length of the database are large. To mitigate the problem, we propose another protocol that can reduce size of shares in DB preparation phase.

We use two parameters *m* and *n* ($m<n$) for computing shares. When $\mathsf {Share}$ outputs $([\![x]\!]_0, [\![x]\!]_1) \in \mathbb {Z}_{2^m}^2$, we denote the share by $([\![x]\!]^m_0, [\![x]\!]^m_1)$. When $\mathsf {Share}$ outputs $([\![x]\!]_0, [\![x]\!]_1) \in \mathbb {Z}_{2^n}^2$, we denote the share by $([\![x]\!]_0, [\![x]\!]_1)$. We denote $M = 2^m$. In our protocol, all the random values are uniformly generated from $\mathbb {Z}_{2^n}$.

*Basic idea* $V_c[i] = \mathsf {LF}_c(i, \hat{T})$ is a lookup table used by Protocol 3 and 4. We sample $V_c[i]$ at $i=0, M, 2M,\ldots , \lfloor N'/M \rfloor M$, where $N'$ is the length of $V_c$ and store the sampled values in a vector *z*. We compute $x[i] = V_c[i] - V_c[p]$ for $i=0,\ldots ,N'-1$, where *p* is the sampled position closest to *i* and $p\le i$. Given a position *k*, we can compute $V_c[k]$ by $z[\lfloor k/M \rfloor ] + x[k]$. Any element in *z* is non-negative and at most $N'-1$ while that in *x* is also non-negative and at most $M-1$ because $0 \le V_c[i+1] - V_c[i] \le 1$. Our idea is to use *n* bits for storing *z*[*i*] and *m* bits for storing *x*[*i*]. Note that we used *n* bits for storing $V_c[i]$ in Protocol 3 and Protocol 4. There are $\lceil N'/M \rceil$ sampled positions, so the size of the lookup table becomes $O(n\lceil N'/M \rceil + mN')$, which is *n*/*m* times smaller compared to $V_c$ if *M* is sufficiently large. We use a rotation technique to hide an intermediate position. Since $1 < V_c[0] - V_c[N'-1]$ for most cases, we design a rotated table $V'$ that satisfies $0 \le {V'}_c[i+1] - {V'}_c[i] \le 1$ by subtracting an offset from $V_c$.

*DB preparation phase* $\mathcal {B}$ computes following vectors for $j=0,\ldots ,\ell -1$

14 $$\begin{aligned} {V'}_{c,f}^j[(i+r_{f}[j])_{\bmod {N'}}] = {\left\{ \begin{array}{ll} V_c[ i ] - o_{c,f}^j &{} ( i \le (i+r_{f}[j])_{\bmod {N'}} )\\ V_c[ i ] - {\bar{o}}_{c,f}^j &{} ( i > (i+r_{f}[j])_{\bmod {N'}}) \;\;, \end{array}\right. } \end{aligned}$$

where $r_{f}[j]$ is a random value, $o_{c,f}^j = V_c[ (N'-1-r_{f}[j])_{\bmod {N'}} ] - V_c[N'-1]$ and ${\bar{o}}_{c,f}^j = V_c[ (N'-1-r_{f}[j])_{\bmod {N'}} ] - V_c[0]$.

### **Theorem 4**

$$0 \le {V'}_{c,f}^j[i+1] - {V'}_{c,f}^j[i] \le 1$$ *for* $$i=0,\ldots ,N'-2$$.

### *Proof*

Following equation is equivalent to Eq. 14.

15 $$\begin{aligned} {V'}_{c,f}^j[i] = {\left\{ \begin{array}{ll} V_c[ (i-r_{f}[j])_{\bmod {N'}} ] - o_{c,f}^j &{} ( (i-r_{f}[j])_{\bmod {N'}} \le i )\\ V_c[ (i-r_{f}[j])_{\bmod {N'}} ] - {\bar{o}}_{c,f}^j &{} ( (i-r_{f}[j])_{\bmod {N'}} > i) \;\;. \end{array}\right. } \end{aligned}$$

$0 \le V_c[i+1] - V_c[i] \le 1$ holds for $i=0,\ldots ,N'-2$ from the definition of $V_c$.

If $(r_{f}[j])_{\bmod {N'}} = 0$, $V_c = {V'}_{c,f}^j$. Therefore, $0 \le {V'}_{c,f}^j[i+1] - {V'}_{c,f}^j[i] \le 1$ holds for $i = 0,\ldots ,N'-2$.

If $(r_{f}[j])_{\bmod {N'}} \ne 0$ and $i=(r_{f}[j]-1)_{\bmod {N'}}$, ${V'}_{c,f}^j[i+1] - {V'}_{c,f}^j[i] = V_c[0] - o_{c,f}^j - V_c[N'-1] + {\bar{o}}_{c,f}^j = 0$. Let us consider when $(r_{f}[j])_{\bmod {N'}} \ne 0$ and $i \ne (r_{f}[j]-1)_{\bmod {N'}}$. We denote $i = (r_{f}[j]-1+a)_{\bmod {N'}} (0<a<N')$. Then, $(i + 1 -r_{f}[j])_{\bmod {N'}} = (a)_{\bmod {N'}}$ and $i+1 = (r_{f}[j]-1+a)_{\bmod {N'}}+1$. Since $(a)_{\bmod {N'}} - ( (r_{f}[j]-1+a)_{\bmod {N'}}+1 ) = (a-1)_{\bmod {N'}} - (r_{f}[j]-1+a)_{\bmod {N'}}$ holds because $0<a$, an offset for ${V'}_{c,f}^j[i+1]$ and that for ${V'}_{c,f}^j[i]$ are same and ${V'}_{c,f}^j[i+1] - {V'}_{c,f}^j[i] = V_c[(a)_{\bmod {N'}}] - V_c[(a-1)_{\bmod {N'}}]$. Therefore, $0 \le {V'}_{c,f}^j[i+1] - {V'}_{c,f}^j[i] \le 1$ holds for $i=0,\ldots ,N'-2$. $\square$

Let $Q_{c,f}^j$ be an integer vector of length $\lceil N'/M \rceil$ such that

$$\begin{aligned} Q_{c,f}^j[p] = {V'}_{c,f}^j[ pM]\;,\;\; \mathrm{and} \;\;\; R_{c,f}^j[i] = {V'}_{c,f}^j[i] - {V'}_{c,f}^j[ M\lfloor i/M \rfloor ]\;. \end{aligned}$$

Note that ${V'}_{c,f}^j[i] = Q_{c,f}^j[ \lfloor i/M \rfloor ] + R_{c,f}^j[i]$, and $V_c[i]$ is obtained by adding an offset to ${V'}_{c,f}^j[i]$.

Since $R_{c,f}^j[i]$ is non-negative and at most $M-1$, $\mathcal {B}$ generates shares $[\![ R_{c,f}^j[i] ]\!]^m$. $\mathcal {B}$ also generates $[\![Q_{c,f}^j[p]]\!]$, $[\![o_{c,f}^j]\!]$, $[\![{\bar{o}}_{c,f}^j]\!]$ and $[\![r_{f}[j]]\!]$. Above shares are used for computing lower bound *f* of an interval. $\mathcal {B}$ generates shares for upper bound *g* in a same manner. Then $\mathcal {B}$ distributes all the shares to $P_0$ and $P_1$.

*Search phase* $\mathcal {A}$ generates table $w$ for a query string *w* by Eq. 11. $\mathcal {A}$ generates shares of *q* and distributes them to $P_0$ and $P_1$. The entire protocol is described in Protocol 5.

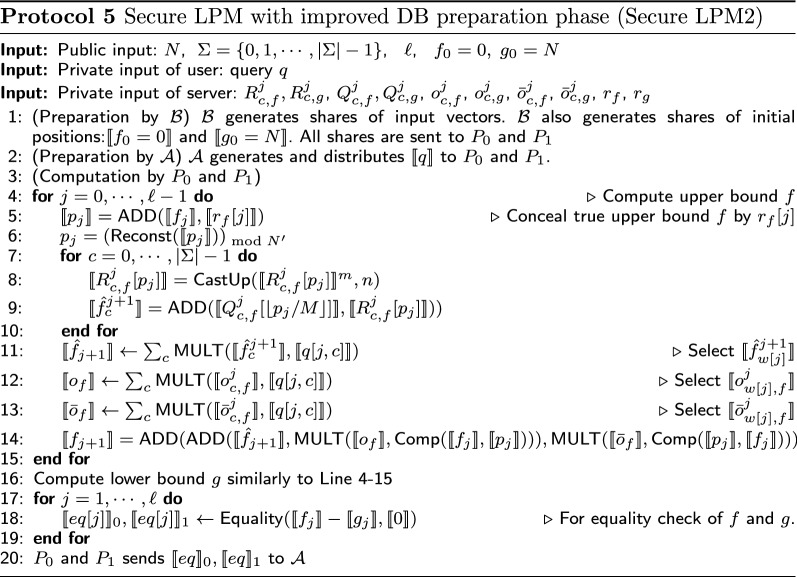



### Security

#### **Theorem 5**

*Protocol 5 is correct and secure in semi-honest setting.*

#### *Proof*

Correctness and security of Protocol 5 are proved as follows.

*Correctness.* In Line 5-6 of Protocol 5, $p_j = (f_j+r_j)_{\bmod {N'}}$ is computed. In Line 8, $\mathsf {CastUp}(R_{c,f}^j[p_j])$ is computed to avoid overflow in Line 9. In Line 9, shares of ${V'}_{c,f}^j[p_j]$ are computed, which is obvious from the definition of $Q_{c,f}^j$ and $R_{c,f}^j$. In Line 11-13, $[\![ {\hat{f}}_{w[j]}^{j+1} ]\!]$, $[\![ o_{{w[j]},f}^{j} ]\!]$ and $[\![ {\bar{o}}_{{w[j]},f}^{j} ]\!]$ are selected. From the definition of ${V'}_{c,f}^j$ described in Eq. 14, it is obvious that $V_c[ f_j ]$ is obtained by ${V'}_{c,f}^j[ p_j ] + o_{c,f}^j$ when $f_j \le p_j$ and ${V'}_{c,f}^j[p_j] + {\bar{o}}_{c,f}^j$ when $f_j > p_j$, and Line 14 computes $[\![ V_c[ f_j ] ]\!]$. *g* is computed similarly to *f*. Since reference to $V_c$ achieved in Lines 4–16 is equivalent to evaluating Eq. 1 and an equality check of $f=g$ is conducted in Lines 17–19, Protocol 5 is correct.

*Security* We only show sketch of the proof. All the messages exchanged between $P_0$ and $P_1$ are shares except for Line 6. In Line 6, reconstructed value $p_j$ is randomized by $r_f[j]$ in Line 5. Therefore, no information is leaked. $\square$

### Complexities

In DB preparation phase, shares of $R^j_{c,f}$ are generated with a parameter *m* and shares of other values including $Q^j_{c,f}$ are generated with a parameter *n*. The length of $R^j_{c,f}$ is $N+1$ and that of $Q^j_{c,f}$ is $\lceil (N+1)/M \rceil$. The total number of other values do not depend on *N*. The query length is $\ell$ and shares of $R^j_{c,f}$, $Q^j_{c,f}$, and other values are necessary for each query character. Therefore, time complexity is $O(\ell N)$ and communication complexity is $O( \ell N m + \ell \lceil N/M \rceil n)$.

For Search phase, $\textsf {ADD}$, $\textsf {MULT}$, $\mathsf {Reconst}$, $\mathsf {CastUp}$ and $\mathsf {Comp}$ are computed a few times for $2\ell$ times in Line 4-16 and $\mathsf {Equality}$ is computed $\ell$ times in Line 17-19. Since each time and communication and round complexities of these subprotocols are *O*(1), those of the entire protocol become $O(\ell )$.

## Experiment

We implemented Protocol 3 (Secure LPM), Protocol 4 (Secure LMEM) and Protocol 5. For comparison, we also implemented baseline protocols (Baseline LPM and Baseline LMEM). Details of the baseline protocols are provided in Appendix 3. All protocols were implemented by Python 3.5.2. The dataset was created from Chromosome 1 of the human genome. We extracted substrings of length $N=10^3$, $10^4$, $10^5$, $10^6$, and $10^7$ for databases, and $\ell =10$, 25, 50, 75, and 100 for queries. $\mathsf {Share}$ was run with $n=16$ and $n=32$ for $N<10^5$ and $10^5 \le N$ in the proposed protocols, and $n=1$ for a Boolean share and $n=8$ for an arithmetic share in the baseline protocols. We did not implement a data transfer module, and each protocol is implemented as a single program. Therefore, the search time of the protocols was measured by the time consumed by either one of $P_0$ and $P_1$. To assess the influence of communication on a realistic environment, we theoretically estimated delays caused by network bandwidth and latency. We assume three environments: LAN (0.2 ms/10 Gbps), WAN$_1$ (10 ms/100 Mbps), and WAN$_2$ (50 ms/10 Mbps). During the run of Search phase, we stored all the data that were transferred from $P_0$ to $P_1$ in a file and measured the file size as an actual communication size. Note that the communication is symmetric and data transfer size from $P_0$ to $P_1$ is equal to that from $P_1$ to $P_0$. Based on the data transfer size *D* byte, we estimate the communication delay by $D/k + eT/1000$, where *k* is bandwidth, *e* is latency and *T* is a round of communication. All the protocols were run with a single thread on the same machine equipped with Intel Xeon 2.2 GHz CPU and 256 GB memory. We also tested the C++ implementation of [19], which is based on AHE. The algorithm for LPM in [17] for the string with $|\Sigma |\le 4$ (e.g., genome sequence) is the same as [19]. Sudo et al. [19] is implemented as a server-client software, and the client and the server were run with individual single threads on the same machine. Therefore, the results of [19] do not include delays caused by bandwidth limitation and latency, so we also estimated delays based on the data transfer size and round of communication in the same manner. Each run of the program was terminated if the total runtime of all phases exceeded 20 h.

**Table 3:** Offline time (Time), offline size (Size), DB preparation time (Time), DB preparation size (Size), Search time on a local machine (Time), Search communication size (Size), estimated Search time for three environments: LAN (0.2 ms/10 Gbps), WAN$_1$ (10 ms/100 Mbps), and WAN$_2$ (50 ms/10 Mbps), for $N=10^4$ (only for Baseline LMEM), $10^5, 10^6, 10^7$, and $\ell =100$

	N	Offline		DB preparation		Search		Estimated timeon network		
		Time	Size	Time	Size	Time	Size	LAN	WAN$_1$	WAN$_2$
Secure	$10^5$	0.166	0.013	123	305	0.141	0.010	0.181	2.162	10.249
LPM	$10^6$	0.141	0.013	1248	3051	0.113	0.010	0.153	2.134	10.221
(proposed)	$10^7$	0.150	0.013	12628	30517	0.126	0.010	0.167	2.147	10.234
Secure	$10^5$	2.318	0.162	123	77	2.888	0.040	3.028	9.911	38.020
LPM2	$10^6$	2.317	0.162	1236	774	2.878	0.040	3.018	9.901	38.010
(proposed)	$10^7$	2.342	0.162	12387	7748	2.939	0.040	3.079	9.962	38.071
	$10^5$	–	–	–	–	691	163	691	707	838
[19]	$10^6$	–	–	–	–	7817	517	7818	7863	8261
	$10^7$	–	–	–	–	20 h<	–	–	–	-
Baseline (LPM)	$10^5$	3995	184	0.146	0.095	13	122	13	24	118
	$10^6$	38767	1841	1.522	0.954	164	1227	165	268	1196
	$10^7$	20 h<	–	–	–	–	–	–	–	–
Secure	$10^5$	7.619	1.704	435	1068	4.817	0.999	5.577	42.900	195.654
LMEM	$10^6$	7.882	1.704	4467	10681	4.926	0.999	5.686	43.009	195.763
(proposed)	$10^7$	8.457	1.704	46384	106811	5.740	0.999	6.501	43.824	196.578
Baseline	$10^4$	12747	611	0.015	0.010	46	407	46	80	389
(LMEM)	$10^5$	20 h<	–	–	–	–	–	–	–	–

The size unit is MB and the time unit is s except for the cell describing “20 h<”

**Fig. 5:**
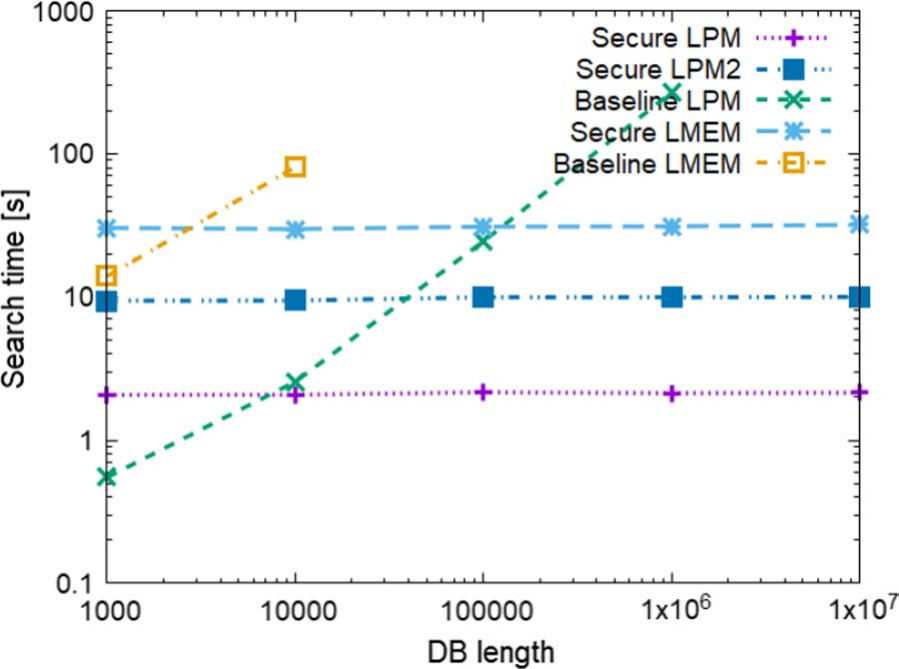
Estimated time (actual search time on a local machine + estimated data-transfer time) for various *N*

**Fig. 6:**
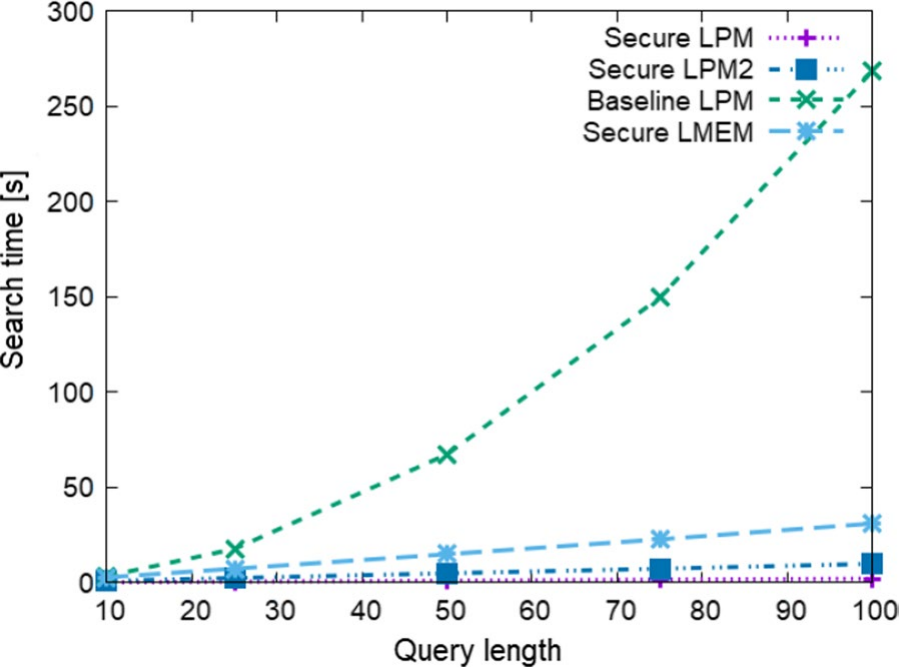
Estimated time (actual search time on a local machine + estimated data-transfer time) for various $\ell$

### Comparison to baseline protocols

Table 3 shows the offline time and size, DB preparation time and size, and Search time and communication size for $N=10^5, 10^6, 10^7$, and $\ell =100$. It also shows the result of Baseline LMEM for $N=10^4$, as the runs for $N>10^4$ did not finish within 20 h. The Search times and communication sizes of Secure LPM and Secure LMEM are several orders of magnitudes faster and smaller than those of Baseline LPM and Baseline LMEM. Since the round and communication complexities of the proposed protocols do not depend on *N*, their estimated Search time remains small even on WAN environments. Figure 5 shows the estimated Search time on WAN$_1$ for $N=10^3, 10^4, \ldots , 10^7$ and $\ell = 100$. The times of Secure LPM and Secure LMEM do not increase, while those of the baseline protocols increase linearly to *N*. Figure 6 shows the estimated Search time on WAN$_1$ for $\ell =10, 25, \ldots , 100$ for $N=10^6$. We can not show the results of Baseline LMEM because none of its runs were finished within the time limit. As shown in the graph, the time of Secure LPM increases linearly to $\ell$ and that of Baseline LPM increases proportionally to $\ell ^2$, which are in good agreement with the theoretical complexities in Table 2. According to the graph, the time of Secure LMEM also increases linearly to $\ell$ though its time and communication complexities are $O(\ell ^2)$. This is because the CPU times are much smaller than the delays caused by network latency that are influenced by the round complexity $O(\ell )$.

We have preliminary results for testing Secure LPM and Baseline LPM on the actual network (10 ms/100 Mbps). The results were 40 s for Secure LPM and 1739 s for Baseline LPM when $N=10^6$. Though both of the preliminary implementations have room for improvement in the performance of data transfer, the results also indicate that our protocol outperforms the baseline protocol and the previous study.

The time and size of Secure LPM and Secure LMEM are several orders of magnitude better than those of the baseline protocols for the offline phase, and vice versa for the DB preparation phase. The total time of the offline and DB preparation phases of our protocols are more than one order magnitude faster than that of baseline protocols. For the total size of the offline and DB preparation phases, Secure LMEM was better than Baseline LMEM, but Baseline LPM was better than Secure LPM though the complexity is better for Secure LPM. This is because the majority of the shares were Boolean in the baseline protocols, while all of the shares were arithmetic in the proposed protocols.

### Comparison to [19]

[19] is a two-party MPC based on AHE. Each homomorphic operation is time consuming and has no offline and DB preparation phases. As shown in Table 3, the Search time of Secure LPM is four orders of magnitude faster than [19] for $N=10^6$. Since time complexity of [19] includes a factor of *N*, the difference in Search time becomes greater as *N* becomes large. Moreover, our protocols have a further advantage in communication for a query response when the network environment is poor, as the round complexity of [19] and our protocols are the same while [19] requires $O(\sqrt{N})$ communication size. The entire runtimes including all the phases are still six times faster for $N=10^5$ and $N=10^6$. We can compute LMEM by examining [19] for all the positions in a query string, but this approach consumed 3406 s and 2.6 GByte of communication for $N=10^4$.

### Result of the approach in section "Reducing size of shares in DB preparation phase"

We also implemented Protocol 5 (Secure LPM2) to investigate a trade-off between reduction of the size of shares in DB preparation phase and increase in search time and communication overhead in Search phase. We used the same programming language (i.e., Python 3.5.2) for the implementation and used the same datasets. $\mathsf {Share}$ was run with $n=8$ when generating the arithmetic shares of *R*. For the generation of rest of the arithmetic shares, $\mathsf {Share}$ was run with $n=16$ and $n=32$ for $N<10^5$ and $10^5 \le N$. (i.e., $m=8$, $n=16$ ($N<10^5$), and $n=32$ ($10^5 \le N$) for the notation used in section "Reducing size of shares in DB preparation phase"). The results are shown in Table 3. The total size of shares in DB preparation phase was 7.7GB for Protocol 5 and 30.5GB for Protocol 3, which is in good agreement with the theoretical complexities discussed in section "Reducing size of shares in DB preparation phase". The search time of Protocol 5 is around 2 s longer than that of Protocol 3. We consider the increase in search time is mainly caused by using rather costly subprotocols: $\mathsf {CastUp}$, $\mathsf {Comp}$ and $\textsf {MULT}$ more times, which also increases the number of communication rounds. Although the increase in search time, Protocol 5 is still more than two orders of magnitude faster than Baseline LPM and three orders of magnitude faster than [19], so we consider that Protocol 5 offers a reasonable trade-off between performance in DB preparation phase and Search phase.

## Discussion

As clearly shown by the results, Search time of the proposed protocols are significantly efficient. Considering the importance of query response time for real applications, it is realistic to reduce Search time at the cost of DB preparation time. Since the total times for offline and DB preparation phases of the proposed protocols were significantly better than those of the well-designed baseline protocols, we consider the trade-off between Search and DB preparation times in our approach to be efficient. For further reduction of DB preparation time, parallelizing the share generation is a feasible approach. Regarding the DB preparation phase, the data transfer between the server and the computing nodes is problematic when the number of queries and the length of the database are large. To mitigate the problem, we also proposed the approach that uses arithmetic shares of a shorter bit length, which offers a reasonable trade-off between the reduction of data size in DB preparation phase and the increase in time and communication overhead in Search phase. Another solution that potentially mitigate the problem is to use an AES-based random number generation that is similar to the technique used in [33]. To explain it briefly, when the server needs to distribute a share of *x*, (1) the server and $P_0$ generate the same randomness *r* using a pre-shared key and a pseudorandom function, and (2) the server computes $x-r$ and sends it to $P_1$. Although $P_0$’s computation cost increases, we can remove the data transfer from the server to $P_0$. In our protocols, the generation of shares in the DB preparation phase cannot be outsourced because they are dependent on the database. Designing an efficient algorithm to outsource the share generation is an important open question.

## Figures and Tables

**Fig. 7:**
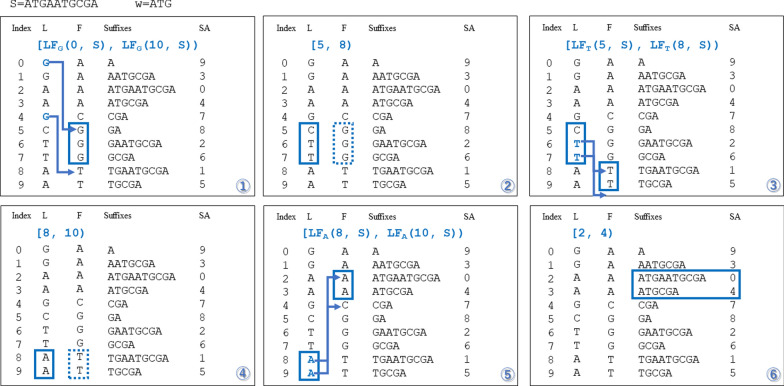
An example of search by FM-Index

**Fig. 8:**
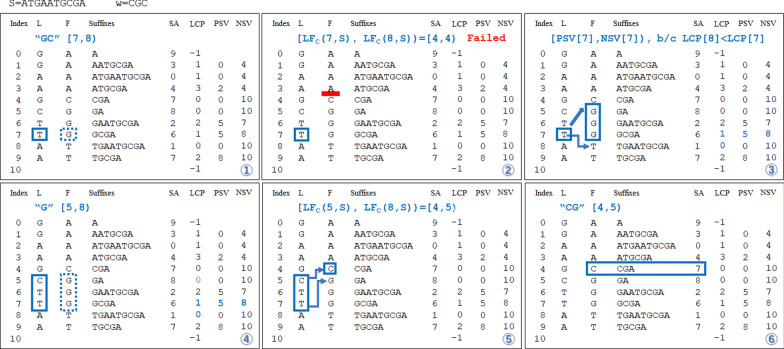
An example of search by FM-ndex, LCP array, PSV and NSV

## Appendices

### Appendix 1: Examples of a aearch with FM-Index and auxiliary data structures

Let us show examples of a search with FM-Index, LCP array, PSV and NSV. In addition to the data structures defined in section "Index structure for string search", we also define a string *F* such that $F[i] = S[ SA[i] ]$. For the case of $S=$ATGAATGCGA, the indices become $SA = ( 9, 3, 0, 4, 7, 8, 2, 6, 1, 5)$, $L =$ GGAAGCTTAA, and $F =$ AAAACGGGTT. Figure 7 illustrates the example of a backward search to find the longest suffix of the query (ATG) that matches the database, and Fig. 8 illustrates the search for MEMs with the query (CGC) by using LCP array, PSV, and NSV. As shown in the upper center panel of Fig. 8, the search failed when the backward search with ‘C’ after finding the interval [7, 8) that corresponds to GC. Since $\mathsf {LCP}[8] \le \mathsf {LCP}[7]$, the parent lcp-interval becomes $[\mathsf {PSV}[7]=5, \mathsf {NSV}[7]=8)$, which corresponds to ‘G’. The match CG is then searched with the backward search with ‘C’ from the parent lcp-interval.

### Appendix 2: Semi-honest security

Here, we recall the simulation-based security notion in the presence of semi-honest adversaries (for two-party computation), as in [32].

#### **Definition 2**

Let $f:(\{0,1\}^*)^2 \rightarrow (\{0,1\}^*)^2$ be a probabilistic 2-ary functionality and $f_i(\vec {x})$ denote the *i*-th element of $f(\vec {x})$ for $\vec {x}=(x_0, x_1)\in (\{0,1\}^*)^2$ and $i\in \{0,1\}$; $f(\vec {x}) = (f_0(\vec {x}), f_1(\vec {x}))$. Let $\Pi$ be a 2-party protocol to compute the functionality *f*. The view of party $P_i$ for $i \in \{0,1\}$ during an execution of $\Pi$ on input $\vec {x}=(x_0, x_1)\in (\{0,1\}^*)$ where $|x_0 | = |x_1 |$, denoted by $\textsc {View}^\Pi _{{i}}(\vec {x})$, consists of $(x_i, r_i, m_{i,1}, \dots , m_{i, t})$, where $x_i$ represents $P_i$’s input, $r_i$ represents its internal random coins, and $m_{i, j}$ represents the *j*-th message that $P_i$ has received. The output of all parties after an execution of $\Pi$ on input $\vec {x}$ is denoted as $\textsc {Output}^\Pi (\vec {x})$. Then, for each party $P_i$, we say that $\Pi$ *privately computes* *f* in the presence of semi-honest corrupted party $P_i$ if there exists a probabilistic polynomial-time algorithm $\mathcal {S}$ such that

$$\begin{aligned} \{(\mathcal {S}(i, x_i, f_i(\vec {x})), f(\vec {x}))\} \equiv \{(\textsc {View}^\Pi _{{i}}(\vec {x}), \textsc {Output}^\Pi (\vec {x}))\}, \end{aligned}$$

where the symbol $\equiv$ means that the two probability distributions are statistically indistinguishable.

As described in [32], the composition theorem for the semi-honest model holds; that is, any protocol is privately computed as long as its subroutines are privately computed.

### Appendix 3: Our secure baseline LPM and LMEM

In this section, we show our secure baseline LCP and LMEM based on secret sharing. We explain how to construct LCP, since we can obtain LMEM by (parallelly) executing LCP for all positions in the query. Note that $\vec {x} = (x_1, x_2, \cdots )$, $\vec {x_i}$ denotes an *i*-th element of $\vec {x}$, $[\![\vec {t}]\!] = ([\![\vec {t}]\!]_0, [\![\vec {t}]\!]_1)$, and $(|\vec {x} |, |\vec {y} |) = (L,N)$. Here, we assume $N > L$. When $[\![\vec {x}]\!] = ([\![x_1]\!], [\![x_2]\!], \cdots , [\![x_p]\!])$, $[\![\vec {x}]\!] \gg 1$ means $([\![0]\!], [\![x_1]\!], \cdots , [\![x]\!]_{p-1})$. In our protocol, we use two subprotocols as follows:

- $\mathsf {All}{} \texttt {-}\mathsf {AND}$ takes a list $[\![\vec {t}]\!]$ (with *p* Boolean shares) as input and outputs $[\![t_1 \wedge \cdots \wedge t_p]\!]^B$. We can compute this function with $\lceil p \rceil$ communication rounds (by appropriate parallelization) and *O*(*p*)-bit data transfer.
- $\mathsf {All}{} \texttt {-}\mathsf {OR}$ takes a list $[\![\vec {u}]\!]$ (with *p* Boolean shares) as input and outputs $[\![u_1 \vee \cdots \vee u_p]\!]^B$. We can compute this function with $\lceil p \rceil$ communication rounds (by appropriate parallelization) and *O*(*p*)-bit data transfer.

Our protocol is as in Protocol A1. In the following, we explain the details of our baseline longest common prefix search protocol using an example that strings $\vec {x} =$ “TGA” and $\vec {y} =$ “ATTGC”. In this example, $w = 2$ since there exists “TG” in $\vec {y}$, but “TGA” does not. For better understanding, we introduce a more straightforward approach and analyze its efficiency before explaining our baseline protocol. In the straightforward approach, we securely check whether the first letter of $\vec {x}$ (i.e., “T”) exists in y or not. Next, we check every pattern up to the second letter of $\vec {x}$ (i.e., “TG”) for a match anywhere in $\vec {y}$. We also execute the same operations for up to the third latter of $\vec {x}$ (i.e., “TGA”). In these processes, we necessary to execute the “check if the characters match”, “check if all the characters match”, and “check if at least one of the perfect matches exist”. For these operations, we need to use *O*(*N*), *O*(*NL*), and *O*(*N*) secure $\textsf {AND}$ gates, respectively. Since we execute these operations for all *L* candidates, the number of $\textsf {AND}$ gates we need for are *O*(*NL*), $O(NL^2)$, and *O*(*NL*), respectively. In these operations, We do not need to compute the letters match for each time since the string is fixed. In our baseline protocol, therefore, we compute whether the letter is matched or not beforehand and repeatedly use them. Since we can check this check with *O*(*NL*), however, our baseline still requires $O(NL^2)$ AND gates. Although it may be possible to reduce the number of AND gates via increasing other costs (e.g., communication rounds), it will not be easy to construct the protocol with *N*-independent online cost like the proposed one with this strategy.

Why the offline cost of our baseline is so significant: In secure computation, it is impossible in principle to change the behavior depending on the computation results in the middle. In other words, we are always forced to perform the worst-case computation. In the previous example, for example, we consider the case for checking whether the first letter of $\vec {x}$ (i.e., “T”) matches any of the letters in y. If it is done in plain text, the moment we find “T” in the second letter of $\vec {y}$, we don’t have to worry about the rest of the letters in $\vec {y}$. In secure computation, however, we have to check everything, including the rest, since we cannot find that the match has already existed. In addition, we consider the case that we check the match for up to the first two letters in $\vec {x}$ (i.e., “TG”) and the first two letters in $\vec {y}$ (i.e., “AT”). In this case, the moment we see A, we can decide there is no match and terminate the process in plaintext computation. In secure computation, however, this is impossible. As we see above, we are always forced to consider the worst-case computing cost in secure computation. Note that offline costs for secure computation are linear to the number of $\textsf {AND}$ gates. We need $O(NL^2)$ offline cost in our baseline (and straightforward) protocol, and *N* is large in our setting. This is why the offline cost of our baseline protocol is so large. Our proposed protocol successfully avoids this problem by developing a new secure primitive and combining it with an appropriate data structure.
